# Genome-wide genetic links between amyotrophic lateral sclerosis and autoimmune diseases

**DOI:** 10.1186/s12916-021-01903-y

**Published:** 2021-02-05

**Authors:** Chun Yu Li, Tian Mi Yang, Ru Wei Ou, Qian Qian Wei, Hui Fang Shang

**Affiliations:** Department of Neurology, Laboratory of Neurodegenerative Disorders, National Clinical Research Center for Geriatric, West China Hospital, Sichuan University, Chengdu, China

**Keywords:** Amyotrophic lateral sclerosis, Autoimmunity, Pleiotropy, Conditional false discovery rate, Genome-wide association study

## Abstract

**Background:**

Epidemiological and clinical studies have suggested comorbidity between amyotrophic lateral sclerosis (ALS) and autoimmune disorders. However, little is known about their shared genetic architecture.

**Methods:**

To examine the relation between ALS and 10 autoimmune diseases, including asthma, celiac disease (CeD), Crohn’s disease (CD), inflammatory bowel disease (IBD), multiple sclerosis (MS), psoriasis, rheumatoid arthritis (RA), systemic lupus erythematosus (SLE), type 1 diabetes (T1D), and ulcerative colitis (UC), and identify shared risk loci, we first estimated the genetic correlation using summary statistics from genome-wide association studies, and then analyzed the genetic enrichment leveraging the conditional false discovery rate statistical method.

**Results:**

We identified a significant positive genetic correlation between ALS and CeD, MS, RA, and SLE, as well as a significant negative genetic correlation between ALS and IBD, UC, and CD. Robust genetic enrichment was observed between ALS and CeD and MS, and moderate enrichment was found between ALS and UC and T1D. Thirteen shared genetic loci were identified, among which five were suggestively significant in another ALS GWAS, namely rs3828599 (*GPX3*), rs3849943 (*C9orf72*), rs7154847 (*G2E3*), rs6571361 (*SCFD1*), and rs9903355 (*GGNBP2*). By integrating *cis*-expression quantitative trait loci analyses in Braineac and GTEx, we further identified *GGNBP2*, *ATXN3*, and *SLC9A8* as novel ALS risk genes. Functional enrichment analysis indicated that the shared risk genes were involved in four pathways including membrane trafficking, vesicle-mediated transport, ER to Golgi anterograde transport, and transport to the Golgi and subsequent modification.

**Conclusions:**

Our findings demonstrate a specific genetic correlation between ALS and autoimmune diseases and identify shared risk loci, including three novel ALS risk genes. These results provide a better understanding for the pleiotropy of ALS and have implications for future therapeutic trials.

**Supplementary Information:**

The online version contains supplementary material available at 10.1186/s12916-021-01903-y.

## Background

Amyotrophic lateral sclerosis (ALS) is a devastating neurodegenerative disorder, characterized by the death of motor neurons as well as paralysis of voluntary muscles [1]. The majority of ALS patients die within 3~5 years after diagnosis, mostly due to respiratory failure [2]. The pathological mechanisms underlying ALS are multifarious and complex, with a sophisticated interaction between genetic and environmental factors [3]. To date, no effective therapies for ALS have been found, and approved drugs only improved survival to a limited extent [4]. Therefore, exploring the pathogenesis of ALS and developing novel therapeutic strategies are necessary and urgent.

Currently, compelling evidence implicates that dysregulated immunities are associated with ALS [5]. It has been established that neuroinflammation is overactivated in ALS, accompanied by microglia transformation, astrocyte proliferation, perivascular infiltration of monocytes and T cells, and dysregulated immune-related genes [6–9]. Typical hallmarks of autoimmunity occurring in the pathogenesis of ALS have also been reported, such as the presence of circulating immune complexes and the evidence of higher frequency of specific histocompatibility types [10]. Moreover, epidemiologic studies presented that several pre-existing autoimmune disorders are associated with an increased risk of ALS [11]. And intermediate alleles of *C9orf72*, the most common genetic cause of ALS, were suggested to be associated with systemic autoimmune diseases, indicating the role of *C9orf72* in immunity regulation [12]. Furthermore, mice harboring loss-of-function mutations in the ortholog of *C9orf72* cause fatal autoimmune diseases [13]. These associations raise the possibility of shared genetic or environmental risk factors, or clues to modifiable triggers that might thereby affect ALS incidence. Therefore, a systematic study is necessary to decipher whether shared polygenic risk variants exist between ALS and autoimmune diseases, and whether specific molecular biological pathways are involved.

Recently, a novel statistical method to investigate genetic overlapping between polygenic traits using summary data from genome-wide association studies (GWAS) have been developed and utilized extensively in several human traits and diseases [14–16]. By incorporating GWAS results from multiple disorders and phenotypes, this method could provide insights into the genetic pleiotropy (defined as a single gene or variant being associated with more than one distinct phenotype) and increased statistical power to discover less significant associations [14–16]. Applying this approach, we systematically evaluated the shared genetic risk between ALS and autoimmune diseases and further conducted functional enrichment analysis.

## Methods

### GWAS summary statistics

We investigated the genetic links between ALS [17] and 10 autoimmune disorders including asthma [18], multiple sclerosis (MS) [19], psoriasis [20], rheumatoid arthritis (RA) [21], systemic lupus erythematosus (SLE) [22], type 1 diabetes (T1D) [23], celiac disease (CeD) [24], inflammatory bowel disease (IBD) [25], Crohn’s disease (CD) [25], and ulcerative colitis (UC) [25] based on GWAS summary statistics. Details of the summary data for all GWAS were shown in Additional file 1: Table S1. The study design including the collection of samples, quality control procedures, and imputation methods have been described in each publication. To confirm the findings in the discovery phase, we further assessed the *P* values of the identified pleiotropic single-nucleotide polymorphisms (SNP) in another ALS GWAS [26]. The relevant institutional review boards or ethics committees approved the research protocol of each GWAS, and all human participants gave written informed consent.

### Statistical analyses

#### Genetic correlation

We estimated the genetic correlation between ALS and each autoimmune disorder using GNOVA [27]. GNOVA estimates genetic covariance with summary data of the genetic variants shared between two GWAS, and then calculates the genetic correlation based on genetic covariance and variant-based heritability. We ran GNOVA on SNPs in both diseases together with reference data derived from the 1000 Genomes Project European population using default parameters. We did not correct for sample overlap when running GNOVA, since no information was available to evaluate the extent of sample overlap between different GWAS. Additionally, considering that different genomic regions disproportionately contributed to the genome-wide correlation, we further quantified the correlation between ALS and each autoimmune disorder in small regions in the genome using ρ-HESS with default parameters [28].

#### Genomic control

Due to population stratification or cryptic relatedness or overcorrection of test statistics [29], the empirical null distribution in GWAS is sometimes inflated or deflated. To correct for such bias, we applied a genomic control method leveraging intergenic SNPs to adjust the summary statistics for each GWAS respectively (Additional file 2) [29–34]. Then, we pruned the SNPs by removing SNPs in linkage disequilibrium (LD) (*r*^2^ > 0.2 within 250 kb) based on 1000 Genomes Project LD structure using plink –clump functionality [34].

#### Pleiotropic enrichment plots

To assess the pleiotropic enrichment, we plotted conditional quantile-quantile plots for ALS by creating subsets of SNPs based on their association with each autoimmune disorder. To further quantitatively assess the level of enrichment, we constructed fold-enrichment plots of nominal -log_10_(*P*) values of ALS for all SNPs and subsets of SNPs determined by the significance of their association with each autoimmune disease (Additional file 2**)** [14–16].

#### Identification of risk loci

To identify risk loci associated with ALS conditional on each autoimmune disease, we computed the conditional false discovery rate (FDR) statistics using the conditional FDR approach (Additional file 2) [14–16]. To reduce false positives, a significance threshold of FDR < 0.01 was utilized. Furthermore, to identify shared risk loci associated with ALS and each autoimmune disease, we computed the conjunctional FDR statistics (Additional file 2) [32, 35, 36]. A significance threshold of FDR < 0.05 was utilized, corresponding to five false positives per 100 reported associations. A stricter threshold was chosen for the conditional statistics since its possibility of false positive was greater. Then, we built the conditional and conjunctional Manhattan plots to illustrate the identified risk loci between ALS and each autoimmune disease (Additional file 2) [14–16]. The annotated gene for each significant locus by ANNOVAR was listed in the plots [33]. For intergenic variants spanning more than 1 gene, the significant variant in each gene will be listed. We used the R implementation of the conditional FDR method available from github.com/KehaoWu/GWAScFDR.

#### Functional evaluation of shared risk loci

To assess whether the shared risk loci modify gene expression, we evaluated *cis*-expression quantitative trait loci (eQTL) in Braineac, a publicly available dataset of normal control brains for investigating the genes and SNPs associated with neurological disorders [37]. We analyzed eQTL for the mean *P* value derived across these brain regions: the cerebellum, frontal cortex, hippocampus, medulla, occipital cortex, putamen, substantia nigra, temporal cortex, thalamus, and white matter. To minimize false positives, a *P* value below 1.0E−04 was considered as significant after Bonferroni correction. Meanwhile, we also analyzed *cis*-eQTL in whole blood, skeletal muscle, and 13 brain tissues (amygdala, anterior cingulate cortex (BA24), caudate basal ganglia, cerebellar hemisphere, cerebellum, cortex, frontal cortex (BA9), hippocampus, hypothalamus, nucleus accumbens basal ganglia, putamen basal ganglia, spinal cord cervical, and substantia nigra) from GTEx v7 [38]. *Cis*-eQTLs as pre-computed by GTEx were downloaded directly from the GTEx portal (http://gtexportal.org/). We applied a nominal *P* value cutoff of 1E−06 to identify significant *cis*-eQTLs, which approximates a false discovery threshold of 0.05.

To identify enrichments in gene ontologic features associated with ALS and autoimmune disorders, we used ConsensusPathDB [39] for functional interaction analysis. The shared risk genes identified with the conjunctional FDR method and eQTL analyses were utilized with default parameters and background gene sets. Biological, cellular, and molecular gene ontologic terms were analyzed. Genes in the HLA region were excluded due to the complex LD patterns.

## Results

### Genetic correlation

We identified a significant positive genetic correlation between ALS and CeD, MS, RA, and SLE, as well as a significant negative genetic correlation between ALS and IBD, UC, and CD (Additional file 1: Table S2). To serve as comparison, we also explored the correlation between Parkinson’s disease (PD) [40], Alzheimer’s disease (AD) [41], and autoimmune diseases using GNOVA with the same pipeline. As a result, significant correlation was only identified between PD and RA after the Bonferroni correction (Additional file 1: Table S2). These results suggested the considerable genetic links between ALS and autoimmune disorders. Furthermore, we searched for genomic regions that disproportionately contributed to the genetic correlation. No regions with significant correlation were identified after Bonferroni correction, while regions with nominally significant correlation were found between ALS and MS, IBD, SLE, CD, UC, and RA (Additional file 1: Figure S1).

### Estimation of pleiotropic enrichment

**Fig. 1:**
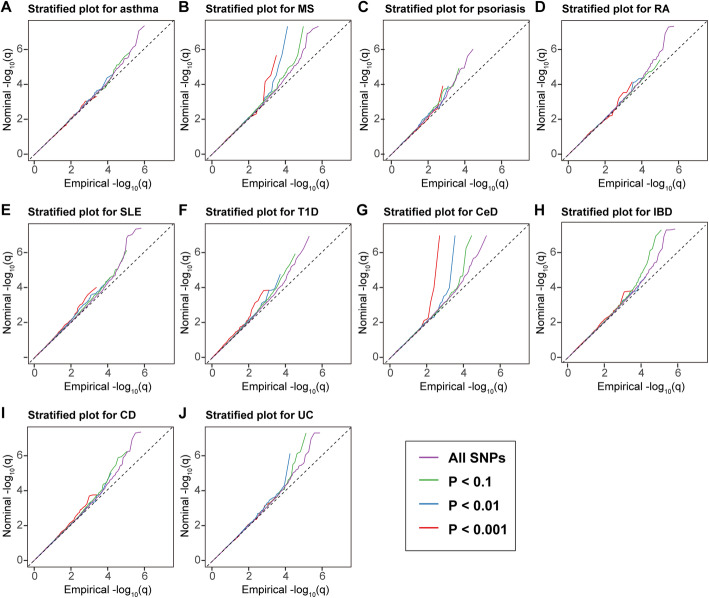
Enrichment plots. Conditional quantile-quantile plots of nominal versus empirical -log_10_(*P*) of ALS as a function of significance of association with autoimmune diseases. Dashed lines indicate the null hypothesis. The figure is suggested to be viewed online for higher resolution

**Fig. 2:**
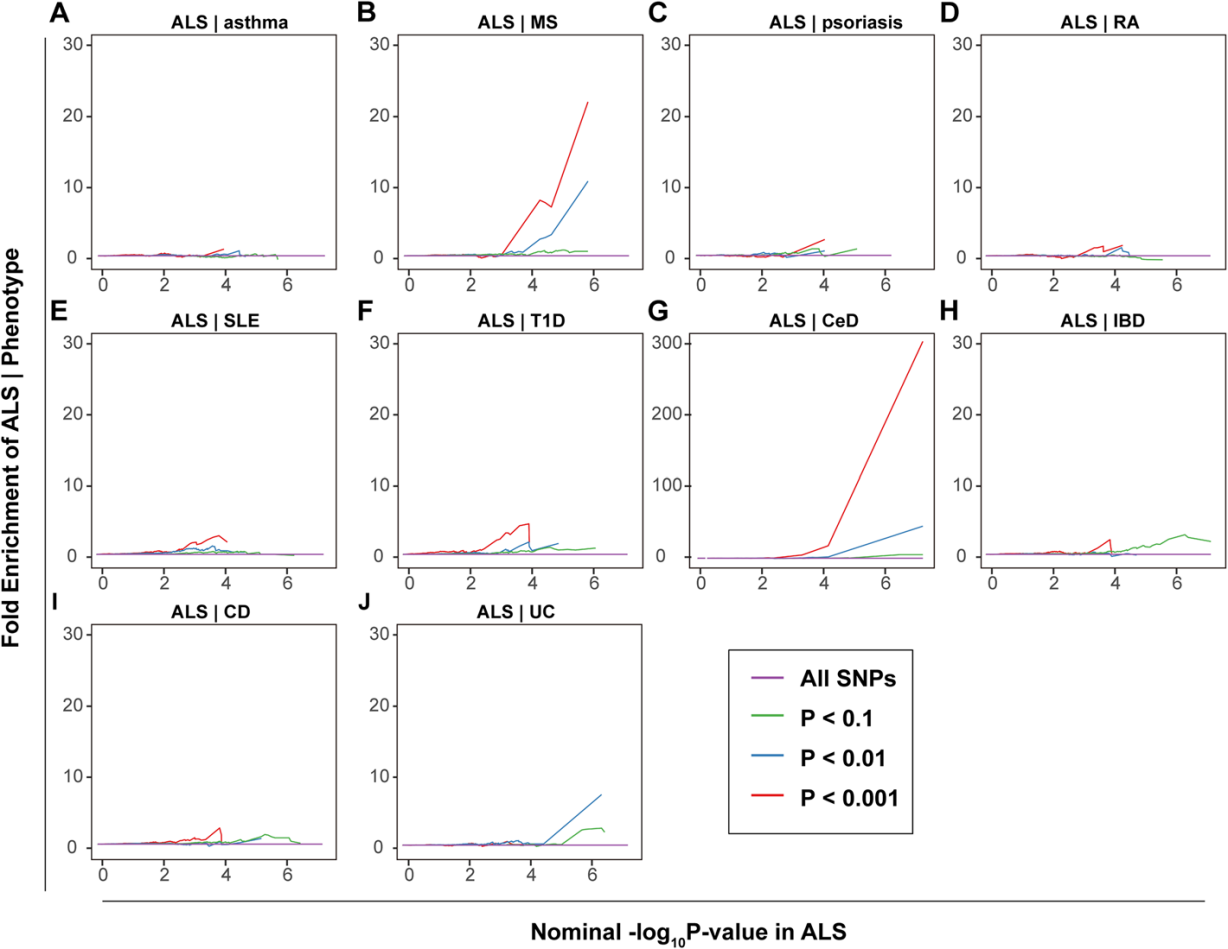
Fold-enrichment plots of nominal -log_10_(*P*) of ALS as a function of significance of association with autoimmune diseases. The figure is suggested to be viewed online for higher resolution

In the stratified quantile-quantile plots for ALS conditional on association *P* values with each autoimmune disease, successive enrichment was found for CeD and MS, and moderate enrichment was found for UC and T1D (Fig. 1), indicating that the proportion of non-null SNPs in ALS increased with higher levels of association with these diseases. In contrast, minimal or no enrichment was found for the other diseases. In the fold-enrichment plots, we could observe over 300-fold enrichment conditional on CeD, approximately 22-fold enrichment on MS, 9-fold enrichment on UC, and 5-fold enrichment on T1D for progressively stringent *P* value thresholds, while minimal enrichment on the other diseases, suggesting a selective genetic overlap between ALS and autoimmune disorders (Fig. 2).

### ALS-associated loci identified with conditional FDR

To discover genetic variants associated with ALS conditional on each autoimmune disease, we performed the conditional FDR statistical analysis. A total of 32 risk loci were identified with conditional FDR < 0.01 (Additional file 1: Table S3, Fig. S2), including 23 novel loci which were not significant (*P* < 5.0E−08) in the original ALS GWAS. Among these 32 loci, 11 were suggestively significant (*P* < 1.0E−05) in the replication GWAS, namely rs3828599 (*GPX3*), rs10463311 (*TNIP1*), rs17070492 (*LOC101927815*), rs9969832 (*MOB3B*), rs2484319 (*C9orf72*), rs12308116 (*C12orf56*), rs447614 (*G2E3*), rs1950882 (*SCFD1*), rs35714695 (*SARM1*), rs2285642 (*GGNBP2*), and rs12608932 (*UNC13A*) (Additional file 1: Table S3). Among these replicated genes, *GPX3*, *TNIP1*, *MOB3B*, *C9orf72*, *SCFD1*, *SARM1*, and *UNC13A* have been described as risk genes for ALS by earlier GWAS, while the others were novel risk genes, including *C12orf56*, *G2E3*, *TRIP11*, and *GGNBP2*.

### Risk loci shared between ALS and autoimmune diseases

**Fig. 3:**
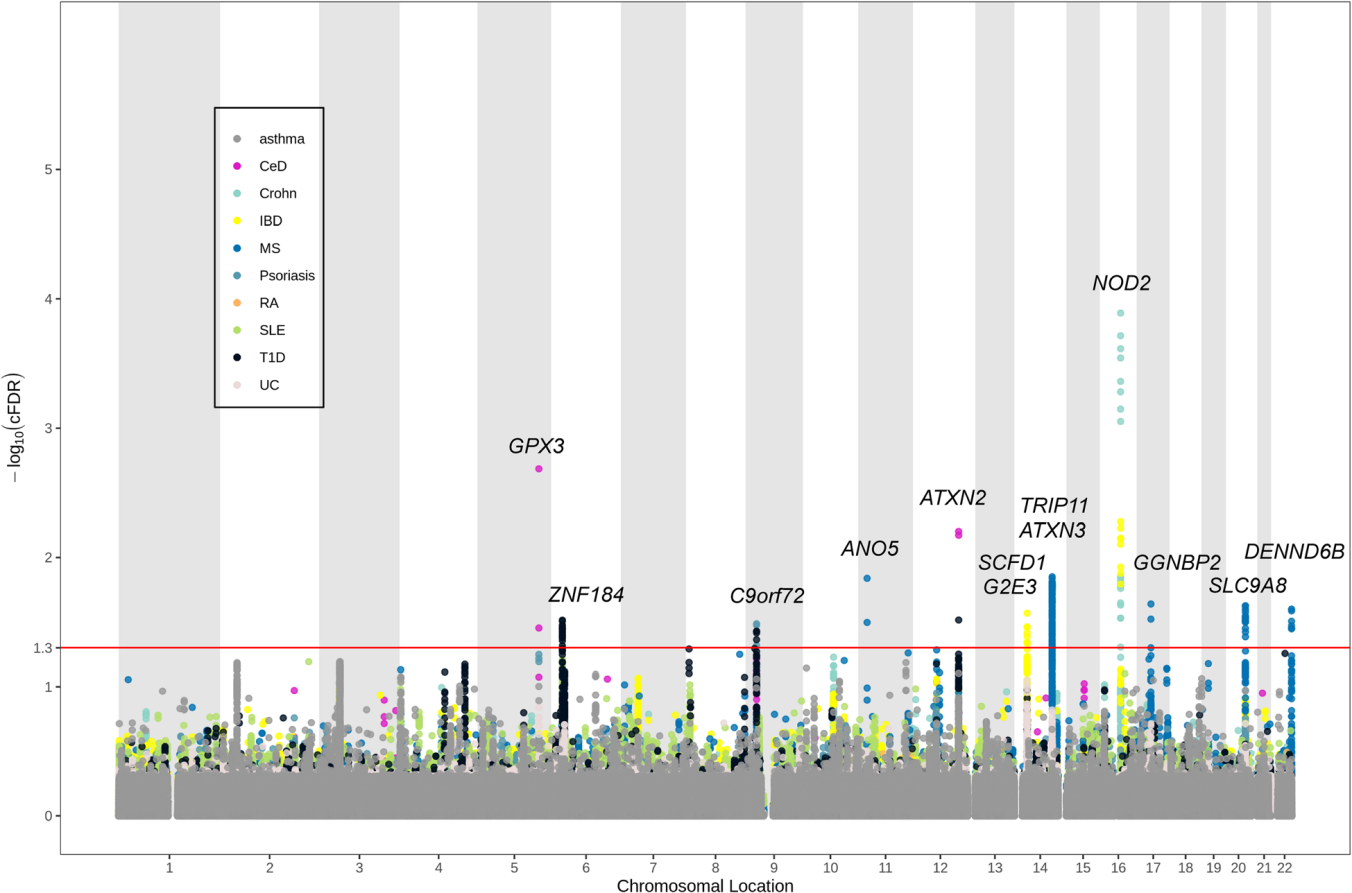
Conjunctional Manhattan plots showing shared genetic loci between ALS and each autoimmune disease. The red horizontal line represents the significant threshold (conjunctional FDR < 0.05)

To identify shared loci between ALS and autoimmune diseases, we further calculated the conjunctional FDR statistics. A total of 13 shared risk loci were identified with conjunctional FDR < 0.05 (Table 1, Fig. 3, Additional file 1: Figure S3). Among the 13 loci, 5 were suggestively significant (*P* < 1.0E−05) in the replication ALS GWAS, namely rs3828599 (*GPX3*), rs3849943 (*C9orf72*), rs7154847 (*G2E3*), rs6571361 (*SCFD1*), and rs9903355 (*GGNBP2*). Among the 5 genes, *G2E3* and *GGNBP2* were newly discovered risk genes for ALS (Table 1). No shared genetic loci were found between ALS and asthma, psoriasis, RA, and SLE, which was consistent with the stratified QQ plots and fold-enrichment plots with no apparent enrichment observed.

**Table 1:** Shared risk loci between ALS and autoimmune disorders

SNP	Genomic position (GRCh37)	Closest gene	FDR value	Associated phenotype	Original ALS *P* value	Replication ALS *P* value
rs3828599	5:150401796	*GPX3*	0.002	CeD	8.08E−08	1.22E−07
rs6456785	6:27390399	*ZNF184*	0.030	T1D	1.23E−04	4.47E−03
rs3849943	9:27543382	*C9orf72*	0.037	T1D	3.77E−30	2.73E−23
rs61880881	11:22270782	*ANO5*	0.015	MS	2.73E−06	n.a.
rs848132	12:111989979	*ATXN2*	0.031	T1D	1.12E−04	2.14E−02
rs7154847	14:31059969	*G2E3*	0.027	IBD	4.46E−07	1.98E−06
rs6571361	14:31183168	*SCFD1*	0.035	IBD	2.54E−07	1.12E−06
rs10138217	14:92497990	*TRIP11*	0.022	MS	1.07E−06	5.26E−05
rs978220	14:92558135	*ATXN3*	0.039	MS	1.66E−06	2.24E−05
rs2076756	16:50756881	*NOD2*	2.44E−04	CD	1.28E−04	n.a.
rs9903355	17:34937221	*GGNBP2*	0.022	MS	2.14E−06	1.01E−06
rs56185963	20:48514826	*SLC9A8*	0.024	MS	8.96E−06	4.53E−04
rs68069258	22:50748930	*DENND6B*	0.026	MS	6.00E−06	n.a.

*SNP* single-nucleotide polymorphism, *A1* effect allele, *FDR* false discovery rate, *n.a.* not available, *CeD* celiac disease, *MS* multiple sclerosis, *T1D* type 1 diabetes, *IBD* inflammatory bowel disease, *CD* Crohn’s disease

### Functional interpretation of shared risk loci

To determine the functional effects of these shared risk loci, we evaluated *cis*-eQTL in Braineac and GTEx. As a result, the pleiotropic risk loci affect the expression of GGNBP2, ATXN3, and SLC9A8 in tissues from both Braineac and GTEx. In addition, the pleiotropic risk loci affect the expression of DCTN4 and TEK in brain regions from Braineac (Table 2), and TRIP11, NOD2, SCFD1, C9orf72, DENND6B, PLXNB2, PPP6R2, DHRS11, MYO19, ZNHIT3, and TMEM116 in tissues from GTEx (Additional file 1: Table S4).

**Table 2:** eQTL revealing functional effects of shared risk SNPs in human brain tissue

Genomic position (GRCh37)	SNP	Closest gene	eQTL	
			Gene	*P* value
17:34937221	rs9903355	*GGNBP2*	*GGNBP2*	8.40E−12
14:92544808	rs11849927	*ATXN3*	*ATXN3*	5.80E−07
20:48586760	rs73274724	*SLC9A8*	*SLC9A8*	7.70E−07
5:150402940	rs8177426	*GPX3*	*DCTN4*	3.10E−05
9:27516640	rs774351	*MOB3B*	*TEK*	3.90E−05

*eQTL* expression quantitative trait loci, *SNP* single-nucleotide polymorphism

To determine the biological pathways represented by shared risk genes and the genes identified with *cis*-eQTL analyses, we conducted pathway over-representation analysis. Four pathways were enriched, namely ER to Golgi anterograde transport (*P* = 5.70E−03), membrane trafficking (*P* = 1.02E−03), transport to the Golgi and subsequent modification (*P* = 8.47E−03), and vesicle-mediated transport (*P* = 1.29E−03). Additionally, 6 GO sets were identified (Additional file 1: Table S5).

## Discussion

In the current study, we investigated the pleiotropy between ALS and autoimmune disorders using summary statistics from large GWAS and the conditional FDR statistical method. We identified significant genetic enrichment for ALS as a function of CeD and MS and moderate enrichment of UC and T1D. Besides, we identified 13 significant shared loci, with 5 validated in the replication ALS GWAS, and 3 were annotated as related to gene expression in tissues from both Braineac and GTEx. These results clarified the shared genetic architecture between ALS and autoimmune diseases, suggested that ALS pathogenesis might be mediated by the dysfunctioning of the immune system, and provided a better understanding for the pleiotropy of ALS.

Neurodegenerative disorders are complex diseases characterized by loss of neurons and axons in the central nervous system. Recently, there is increasing recognition that inflammation and immune response play important roles in the pathogenesis of neurodegeneration [42]. Activated microglia, which is a key regulator of brain inflammation, is also an important cause for neuronal loss in models of neurodegenerative diseases [43]. Previous studies have investigated pleiotropy between AD, PD and autoimmune diseases [15, 16], and identified shared risk genes. However, the pleiotropy between ALS and autoimmune disorders is still not clear. Our results supported the hypothesis of shared genetic risk between ALS and autoimmune diseases and supplemented current knowledge for the correlation between neurodegeneration and autoimmunity.

We observed a substantial genetic enrichment between CeD and ALS. CeD is an autoimmune disorder that occurs in genetically predisposed individuals who develop an immune reaction to gluten [44]. Meanwhile, gluten sensitivity has also been shown to induce neurologic manifestations [45]. For example, a recent case-control designed study suggested that ALS might be associated with autoimmunity and gluten sensitivity [44]. However, such links still need to be confirmed further, as subsequent studies reported inconsistent results [46]. We also identified a shared risk gene *GPX3*, which could have functional relevance to both diseases. *GPX3* is an antioxidant molecule functionally related to *SOD1* [47], the first causative gene for ALS. In a mass spectrometric screen of sera of *SOD1*^H46R^ rats compared to wild-type controls, *GPX3* expression was increased by 1.3 fold in the pre-symptomatic stage, while decreased by 0.74 fold in the end stage of the disease [48]. Meanwhile, *GPX3* activity was reduced in CeD patients (*P* < 0.001) in a recent pilot study, suggesting its potential role in the pathogenesis of CeD [49]. In contrast, minimal enrichment was observed between ALS and CD, which has partially shared genetic basis and pathogenesis with CeD [50]. However, the strongest signal rs2076756 (*NOD2*) in the conjunctional Manhattan plot was identified between ALS and CD. *NOD2* is a member of the pattern-recognition receptor family and can recognize muramyl dipeptide in cytomembrane to activate the NF-κB pathway and stimulate inflammatory factor response [51]. Recently, *NOD2* was identified as a potent autophagy inducer as well, suggesting its potential role in neurodegeneration [52]. In addition, moderate enrichment was observed between ALS and UC, and *G2E3*-*SCFD1* was identified as shared risk genes between ALS and IBD. These results together suggested a potential link between ALS and chronic inflammation of the gastrointestinal tract, though such link was not consistently detected based on current results. Further explorations are warranted to elucidate the correlation.

We also observed genetic enrichment between ALS and MS. Both ALS and MS are complex neurological disorders with sophisticated interactions of environmental toxicity and genetic predisposition [53]. By far, the origin of MS and ALS is still unknown, but the progressive central axonal degeneration is seen in both MS and ALS. And there is evidence suggesting shared cellular mechanisms affecting the disease progression, particularly glial responses in the two disorders [54]. One explanation would be that the two conditions share some common genes which predispose to both MS and ALS. Here, we identified six shared risk genes including *TRIP11*, *ATXN3*, *GGNBP2*, *SLC9A8*, *DENND6B*, and *ANO5* (Table 1). The six shared genes were enriched in two significant cellular components, namely organelle subcompartment (GO:0031984, *P* = 0.0008) and endomembrane system (GO:0012505, *P* = 0.0032) based on results from ConsensusPathDB. Therefore, the membrane system may implicate the overlapping factors related to MS and ALS, and the shared genes might be responsible for the link.

Association between ALS and diabetes has been observed in epidemiological studies [55, 56]. Recently, a causal protective role of type 2 diabetes on ALS was noticed in the European population [57]. In contrast, the association between T1D and ALS was still less understood, and T1D might increase the risk for ALS based on a recent retrospective population-based study [11, 56], though such results were still awaiting further replications [58]. In our study, we observed enrichment between ALS and T1D, and identified three shared risk genes *C9orf72*, *ZNF184*, and *ATXN2*. Repeat expansions in *C9orf72* is a frequent cause of ALS, and *C9orf72* carriers tend to have autoimmune diseases more frequently, suggesting autoimmune inflammation may be intrinsically linked to ALS pathophysiology [59]. Meanwhile, a recent study found that mutations disrupting the normal function of *C9orf72* cause mice to develop features of autoimmunity [12]. Thus, *C9orf72* might serve as an important factor related to inflammation and autoimmunity [12]. *ZNF184* is in the extended major histocompatibility complex (MHC) region, which plays a complex but important role in both neurodegenerative and autoimmune diseases. *ATXN2* has been reported to be associated with several autoimmune diseases like T1D [60], CD [61], and CeD [62] by GWAS. Meanwhile, high-length repeats of CAG trinucleotide in *ATXN2* was identified as a risk factor for ALS as well [63, 64]. Taken together, genetic correlation exists between ALS and T1D, and genes such as *C9orf72*, *ZNF184*, and *ATXN2* might function as potential links between the two diseases.

Compared with CeD, MS, T1D, and UC, we did not detect enrichment between ALS and RA, psoriasis, asthma, CD, and SLE. The results were to a large extent in agreement with a previous epidemiologic study which investigated whether ALS incidence was higher in people with prior autoimmune diseases [11]. This study found that patients with CeD, younger-onset diabetes, MS, asthma, and SLE were at higher risk of ALS, and the rate ratio for UC was borderline significant (*P* = 0.05), and no significant difference was found for RA, psoriasis and CD. As for the different results for asthma and SLE between our study and this epidemiological study, further explorations were warranted to elucidate their correlation with ALS. Moreover, we noted that both positive and negative genetic correlations were observed between ALS and autoimmune disorders (Additional file 1: Table S2). We identified a significant and positive genetic correlation between ALS and CeD, MS, RA, and SLE, indicating that the direction of effect of risk-increasing and protective alleles is consistently aligned between ALS and these autoimmune disorders at genome wide. In contrast, we found a significant and negative genetic correlation between ALS and IBD, UC, and CD, implying these diseases may possess divergent biological mechanisms from the other autoimmune disorders. Such results were in line with previous epidemiological research, which detected no significant difference in the incidence rate of ALS among patients with prior UC and CD compared with controls [11]. Additionally, IBD, including UC and CD, was characterized by chronic inflammation of the gastrointestinal tract, which fulfilled some of the criteria required for classification as autoimmune disorders, while the extent of the involvement remained to be determined [65]. Taken together, immune and autoimmune mechanisms are complex and may vary from disease to disease, so a deeper investigation into the molecular mechanisms involved in these diseases is necessary to further understand the correlation.

Combining cFDR analyses results and eQTL analyses results from Braineac and GTEx, we identified three risk genes, namely *SLC9A8*, *ATXN3*, and *GGNBP2*. *SLC9A8* is a kind of sodium-hydrogen exchangers (NHEs) that exchange extracellular Na^+^ for intracellular H^+^. Ionic homeostasis dysregulation has been proposed as the main trigger of the pathological cascade which brings to motor-neuron loss [66]. A recent study also observed a marked increase in the persistent component of the Na^+^ current from pre-symptomatic SOD1^G93A^ mice [67, 68], suggesting the potential role of *SLC9A8* in ALS etiology. *ATXN3* provides instructions for making an enzyme called ataxin-3, which is involved in destroying excessive or damaged proteins. A trinucleotide repeat expansion in *ATXN3* could cause spinocerebellar ataxia type 3, a neurologic disorder that is characterized by progressive ataxia. Mutations in *ATXN3* affect neurons and other types of brain cells and alter transcription of multiple signal transduction pathways including depressed Wnt and elevated growth factor pathways [69]. Moreover, GWAS has identified suggestively significant SNPs in *ATXN3* as associated with ALS [17]. These findings suggest the potential involvement of *ATXN3* in ALS. Previous studies have suggested the potential role of *GGNBP2* in ALS through gene-based association analysis and summary statistics-based Mendelian randomization (SMR) analysis [26, 70]. Meanwhile, we noticed that *GGNBP2* is an important tumor suppresser involved in several kinds of cancers [71]. Cancer and neurodegenerative diseases are like the two sides of a coin. The pathways that cause neuronal apoptosis, like mitogen-activated protein kinase (MAPK) signaling, can cause uncontrolled neuronal growth as well. Epidemiological studies have shown that the overall risk of cancer was significantly reduced in patients with ALS [72]. Therefore, *GGNBP2* might act as potential genetic links between ALS and cancer.

Using the pleiotropic genes for functional enrichment analysis in ConsensusPathDB, we identified four enriched pathways, all of which were somehow involved in the pathogenesis of ALS. Membrane trafficking has been implicated in virtually every aspect of neuronal function, particularly neuronal maintenance and degeneration [73]. Intracellular membrane trafficking defects affecting key neuronal functions may be an early determinant of motor neuron loss in ALS [74]. Meanwhile, the molecular regulation of intracellular and extracellular vesicle trafficking is an important pathway in ALS pathogenesis [75], and mutation in the vesicle-trafficking protein has been implicated to cause late-onset spinal muscular atrophy and ALS. Fragmentation of the Golgi apparatus has been detected in motor neurons of ALS patients and animal models [76], and mutant *SOD1* was shown to inhibit secretory protein transport from the ER to Golgi apparatus [77].

Combining results from previous publications that estimate pleiotropy between AD, PD and autoimmune diseases using the conditional FDR method, we found that all three neurodegenerative disorders were strongly or moderately enriched on immune-mediated enteropathy like CeD, CD, and UC. Gut microbiota has been shown to play an essential role in IBD and CeD [78], while in recent years, gut microbiome alterations were found to be closely related to neurodegeneration as well [79]. The microbiota-gut-brain axis composed of endocrinological, immunological, and neural mediators was even proposed due to its involvement in neurodegenerative diseases [80]. Our pleiotropic analyses together with previous studies further emphasized that gut microbiota plays an important role in the pathogenesis of neurodegenerative disorders. In addition, differential enrichment was also observed between autoimmune disorders and AD, PD, and ALS respectively. The diversity suggested the variation between the pathogenesis of different neurodegenerative disorders.

### Strengths and limitations

Using the pleiotropy-based statistical method, we identified novel significant SNPs associated with ALS and autoimmune disorders. These SNPs were mostly suggestively significant in the original studies but overlooked due to the limitation of the current GWAS sample size. Moreover, the current results have clinical implications. Since we combined GWAS summary statistics from different but related diseases, the findings may increase our understanding of the pathogenetic mechanisms influenced by pleiotropic loci and facilitate novel treatment strategies in clinical trials. However, the GWAS used in the current study were mostly performed on participants of European ancestry; thus, the findings of shared genetic architecture might be biased and not applicable to other populations. Meanwhile, there was a potential sample overlap between each GWAS. Such overlap might bring some bias to the statistical analysis, although the bias will be minimal. Meanwhile, there was sample overlap between the discovery ALS GWAS and replication ALS GWAS. To strengthen the validity of the identified pleiotropic SNPs, we utilized a more stringent significance threshold (*P* = 1E−05) instead of the commonly used threshold in replication (*P* = 0.05). In addition, the sample size for some diseases like CeD and SLE was relatively smaller, which might influence the results to some extent. Besides, although we identified several pleiotropic SNPs, how these SNPs or genes involved in the pathogenesis of ALS and autoimmune diseases were still little understood. Further functional explorations will provide better understandings.

## Conclusions

By integrating GWAS summary data and the conditional FDR statistical method, we identified selective pleiotropy and novel shared loci between ALS and autoimmune diseases. We further identified novel ALS risk genes *SLC9A8*, *ATXN3*, and *GGNBP2* by combining eQTL analyses. These findings could provide novel insights into the shared genetic background between ALS and autoimmunity and help better understand the etiology of ALS.

## Supplementary Information

**Additional file 1: Table S1.** Summary data from all GWAS used in current study. **Table S2.** Genetic correlation between AD, ALS, PD and autoimmune disorders. **Table S3.** Risk loci associated with ALS conditional on autoimmune diseases. **Table S4.** eQTL revealing functional effects of shared risk SNPs in tissues from GTEx. **Table S5.** Functional annotation of shared risk genes. **Figure S1.** Manhattan-style plots showing the estimates of local genetic correlation between ALS and autoimmune disorders. **Figure S2.** Conditional Manhattan plots showing risk loci for ALS conditional on each autoimmune disease. **Figure S3.** Regional association plots for shared risk loci between ALS and each autoimmune disease

**Additional file 2.** Supplementary Methods.

## Data Availability

The datasets used and/or analyzed during the current study are available from the corresponding author on reasonable request. Code or algorithm used to generate results in this study is available from the corresponding authors on reasonable request.

## Abbreviations

ALS: Amyotrophic lateral sclerosis
GWAS: Genome-wide association study
SNP: Single-nucleotide polymorphism
LD: Linkage disequilibrium
FDR: False discovery rate
eQTL: Expression quantitative trait loci
GO: Gene ontology
SMR: Summary statistics-based Mendelian randomization
MS: Multiple sclerosis
SLE: Systemic lupus erythematosus
T1D: Type 1 diabetes
CeD: Celiac disease
CD: Crohn’s disease
IBD: Inflammatory bowel disease
UC: Ulcerative colitis
PD: Parkinson’s disease
AD: Alzheimer’s disease
