# Supplementary material for: Genome-wide genetic links between amyotrophic lateral sclerosis and autoimmune diseases
Source: BMC Med. 2021 Feb 5;19:27. doi: 10.1186/s12916-021-01903-y (PMC7863260; doi:10.1186/s12916-021-01903-y)
Supplement: Supplementary file 1 — Additional file 1: Table S1. Summary data from all GWAS used in current study. Table S2. Genetic correlation between AD, ALS, PD and autoimmune disorders. Table S3. Risk loci associated with ALS conditional on autoimmune diseases. Table S4. eQTL revealing functional effects of shared risk SNPs in tissues from GTEx. Table S5. Functional annotation of shared risk genes. Figure S1. Manhattan-style plots showing the estimates of local genetic correlation between ALS and autoimmune disorders. Figure S2. Conditional Manhattan plots showing risk loci for ALS conditional on each autoimmune disease. Figure S3. Regional association plots for shared risk loci between ALS and each autoimmune disease [file 12916_2021_1903_MOESM1_ESM.pdf]

# Additional file 1: supplementary figures and tables

**Table S1. Summary data from all GWAS used in current study**

| Disease | Abbreviation | Cases | Controls | Ethnics | SNPs | Reference |
|---|---|---|---|---|---|---|
| Amyotrophic lateral sclerosis | ALS | 20,806 | 59,804 | EUR | 10,031,417 | 17 |
| Amyotrophic lateral sclerosis | ALS replication | 14,387 | 27,008 | EUR & EAS | 6,613,543 | 26 |
| asthma | asthma | 10,549 | 47,146 | EUR | 33,771,858 | 18 |
| Celiac disease | CeD | 4,533 | 10,750 | EUR | 523,398 | 24 |
| Crohn's disease | CD | 12,194 | 28,072 | EUR | 9,570,787 | 25 |
| inflammatory bowel disease | IBD | 25,042 | 34,915 | EUR | 7,345,446 | 25 |
| multiple sclerosis | MS | 33,647 | 449,056 | EUR | 17,510,617 | 19 |
| psoriasis | psoriasis | 17,008 | 37,154 | EUR | 7,055,881 | 20 |
| rheumatoid arthritis | RA | 29,880 | 73,758 | EUR & EAS | 9,739,303 | 21 |
| systemic lupus erythematosus | SLE | 7,219 | 15,991 | EUR | 7,915,251 | 22 |
| type 1 diabetes | T1D | 9,934 | 16,956 | EUR | 2,060,920 | 23 |
| ulcerative colitis | UC | 12,366 | 33,609 | EUR | 9,588,016 | 25 |
| Parkinson's disease | PD | 33,674 | 449,056 | EUR | 17,510,617 | 36 |
| Alzheimer's disease | AD | 17,008 | 37,154 | EUR | 7,055,881 | 37 |

EUR, European; EAS, East Asian; SNP, single nucleotide polymorphism; GWAS, genome-wide association study; 23andMe contributions were excluded in the PD GWAS, as described in the original publication.

**Table S2. Genetic correlation between AD, ALS, PD and autoimmune disorders**

| | AD | ALS | PD |
|---|---|---|---|
| asthma | 0.04 (0.45) | 0.08 (0.23) | -0.10 (0.01) |
| CeD | -0.03 (0.54) | **0.58 (5.82E-28)** | 0.05 (0.24) |
| CD | -0.02 (0.48) | **-0.25 (2.75E-10)** | 0.00 (0.88) |
| IBD | -0.02 (0.55) | **-0.33 (1.16E-16)** | -0.02 (0.41) |
| MS | 0.05 (0.18) | **0.23 (1.11E-08)** | 0.02 (0.62) |
| psoriasis | 0.07 (0.29) | -2.86E-03 (0.97) | -0.02 (0.82) |
| RA | 0.03 (0.53) | **0.22 (6.17E-07)** | **-0.10 (3.31E-03)** |
| SLE | 0.03 (0.36) | **0.26 (6.58E-07)** | 0.04 (0.26) |
| UC | -0.01 (0.68) | **-0.32 (1.96E-13)** | -0.06 (0.09) |

Values are presented as genetic correlation (P value). Values in bold denote significant correlation.
Genetic correlation for T1D was not estimated since beta value was not available in the original GWAS.

**Table S3. Risk loci associated with ALS conditional on autoimmune diseases**

| SNP | Genomic position (GRCh37) | Closest gene | FDR value | Original ALS P value | Replication ALS P value |
|---|---|---|---|---|---|
| rs72714928 | 1:161207232 | *NR1I3* | 5.20E-03 | 2.12E-06 | 4.29E-04 |
| rs1004197 | 1:94249540 | *BCAR3* | 3.36E-03 | 1.22E-06 | 2.83E-02 |
| rs61527579 | 2:76237892 | *GCFC2* | 5.27E-03 | 2.67E-06 | 1.22E-03 |
| rs3828599 | 5:150401796 | *GPX3* | 3.26E-05 | 8.08E-08 | 1.22E-07 |
| rs10463311 | 5:150410835 | *TNIP1* | 1.72E-04 | 4.00E-08 | 1.32E-08 |
| rs538622 | 5:172347679 | *ERGIC1* | 2.00E-03 | 6.69E-07 | 1.22E-04 |
| rs7805982 | 7:42429859 | *GLI3* | 6.56E-03 | 2.86E-06 | 2.35E-04 |
| rs17070492 | 8:2420855 | *LOC101927815* | 5.33E-04 | 1.04E-07 | 3.27E-07 |
| rs9969832 | 9:27493063 | *MOB3B* | 3.39E-24 | 2.01E-28 | 5.03E-21 |
| rs2484319 | 9:27563755 | *C9orf72* | 1.86E-24 | 1.27E-29 | 5.74E-23 |
| rs11195948 | 10:114163515 | *ACSL5* | 2.26E-03 | 9.14E-07 | 4.04E-05 |
| rs61880881 | 11:22270782 | *ANO5* | 2.25E-03 | 2.73E-06 | n.a. |
| rs653178 | 12:112007756 | *ATXN2* | 6.73E-03 | 5.18E-04 | n.a. |
| rs118082508 | 12:57318819 | *SDR9C7* | 1.60E-04 | 1.97E-08 | n.a. |
| rs113247976 | 12:57975700 | *KIF5A* | 2.60E-06 | 6.43E-10 | n.a. |
| rs12320537 | 12:58021091 | *B4GALNT1* | 5.74E-03 | 6.97E-06 | 7.59E-02 |
| rs116900480 | 12:58656105 | *LINC02403* | 5.34E-06 | 6.60E-10 | n.a. |
| rs12308116 | 12:64710719 | *C12orf56* | 6.50E-03 | 4.32E-06 | 6.11E-07 |
| rs41292019 | 12:64849716 | *TBK1* | 8.12E-05 | 8.39E-09 | n.a. |
| rs447614 | 14:31080799 | *G2E3* | 1.24E-03 | 1.74E-07 | 3.96E-07 |
| rs1950882 | 14:31172253 | *SCFD1* | 1.22E-03 | 1.69E-07 | 7.69E-07 |
| rs2295172 | 14:92492651 | *TRIP11* | 2.65E-03 | 8.91E-07 | 1.38E-04 |
| rs1107115 | 14:92546544 | *ATXN3* | 1.93E-03 | 6.47E-07 | 4.38E-05 |
| rs62029863 | 16:50749408 | *NOD2* | 7.12E-04 | 7.91E-05 | n.a. |
| rs34517613 | 17:26610252 | *KRT18P55* | 5.31E-03 | 2.18E-06 | n.a. |
| rs35714695 | 17:26719788 | *SARM1* | 8.25E-03 | 2.89E-06 | 3.07E-10 |
| rs2285642 | 17:34912744 | *GGNBP2* | 4.52E-03 | 1.80E-06 | 1.18E-06 |
| rs12608932 | 19:17752689 | *UNC13A* | 3.86E-11 | 6.35E-15 | 2.70E-10 |
| rs56185963 | 20:48514826 | *SLC9A8* | 8.34E-03 | 8.96E-06 | 4.53E-04 |
| rs112572132 | 20:48533630 | *LOC105372653* | 5.35E-03 | 6.50E-06 | 4.40E-04 |
| rs75087725 | 21:45753117 | *CFAP410* | 2.17E-10 | 1.85E-14 | n.a. |
| rs62241220 | 22:50747507 | *DENND6B* | 7.37E-03 | 7.45E-06 | n.a. |

n.a., not available; SNP, single nucleotide polymorphism; FDR, false discovery rate.

**Table S4. eQTL revealing functional effects of shared risk SNPs in tissues from GTEx**

| Gene | Tissue | Genomic position (GRCh37) | SNP | Nominal P value |
|---|---|---|---|---|
| *ATXN3* | Brain_Cerebellum | 14:92495536 | rs2896190 | 2.14E-08 |
| *ATXN3* | Muscle_Skeletal | 14:92541852 | rs7152876 | 1.25E-10 |
| *C9orf72* | Brain_Nucleus_accumbens_basal_ganglia | 9:27559835 | rs774357 | 2.71E-08 |
| *C9orf72* | Brain_Spinal_cord_cervical_c-1 | 9:27516640 | rs774351 | 7.62E-07 |
| *C9orf72* | Muscle_Skeletal | 9:27516640 | rs774351 | 7.63E-08 |
| *DENND6B* | Brain_Cortex | 22:50748930 | rs68069258 | 5.06E-10 |
| *DENND6B* | Brain_Frontal_Cortex_BA9 | 22:50742935 | rs34544325 | 8.60E-10 |
| *DENND6B* | Whole_Blood | 22:50742611 | rs62241211 | 3.33E-08 |
| *DHRS11* | Brain_Cerebellum | 17:34951204 | rs2285640 | 2.54E-07 |
| *DHRS11* | Brain_Cortex | 17:34937221 | rs9903355 | 1.31E-09 |
| *DHRS11* | Brain_Frontal_Cortex_BA9 | 17:34937221 | rs9903355 | 8.11E-07 |
| *DHRS11* | Brain_Nucleus_accumbens_basal_ganglia | 17:34951204 | rs2285640 | 9.76E-09 |
| *DHRS11* | Muscle_Skeletal | 17:34951204 | rs2285640 | 2.74E-10 |
| *GGNBP2* | Brain_Caudate_basal_ganglia | 17:34937221 | rs9903355 | 7.94E-08 |
| *GGNBP2* | Muscle_Skeletal | 17:34951204 | rs2285640 | 3.60E-14 |
| *GGNBP2* | Whole_Blood | 17:34951204 | rs2285640 | 5.64E-11 |
| *MYO19* | Muscle_Skeletal | 17:34951204 | rs2285640 | 1.64E-21 |
| *MYO19* | Whole_Blood | 17:34951204 | rs2285640 | 7.15E-09 |
| *NOD2* | Whole_Blood | 16:50730229 | rs2066850 | 7.84E-11 |
| *PLXNB2* | Muscle_Skeletal | 22:50742935 | rs34544325 | 5.05E-21 |
| *PPP6R2* | Muscle_Skeletal | 22:50747507 | rs62241220 | 9.39E-16 |
| *SCFD1* | Brain_Anterior_cingulate_cortex_BA24 | 14:31183168 | rs6571361 | 2.03E-07 |
| *SCFD1* | Brain_Cerebellar_Hemisphere | 14:31059969 | rs7154847 | 2.52E-08 |
| *SCFD1* | Brain_Cerebellum | 14:31183168 | rs6571361 | 1.25E-12 |
| *SCFD1* | Muscle_Skeletal | 14:31080800 | rs447853 | 1.70E-11 |
| *SCFD1* | Whole_Blood | 14:31183168 | rs6571361 | 2.36E-25 |
| *SLC9A8* | Brain_Cerebellar_Hemisphere | 20:48578734 | rs113558364 | 1.36E-10 |
| *SLC9A8* | Brain_Cerebellum | 20:48561156 | rs4810992 | 3.10E-08 |
| *SLC9A8* | Muscle_Skeletal | 20:48561156 | rs4810992 | 9.97E-13 |
| *TMEM116* | Whole_Blood | 12:111989979 | rs848132 | 4.42E-07 |
| *TRIP11* | Brain_Cerebellum | 14:92496613 | rs4556729 | 4.48E-07 |
| *TRIP11* | Muscle_Skeletal | 14:92497990 | rs10138217 | 3.96E-18 |
| *ZNHIT3* | Whole_Blood | 17:34937221 | rs9903355 | 8.23E-15 |

cis-eQTL with the most significant nominal P value for each gene in target tissues were listed

**Table S5. Functional annotation of shared risk genes**

| GO ID | | P value | FDR adjusted P value |
|---|---|---|---|
| GO:0097517 | contractile actin filament bundle | 1.26E-03 | 0.00378 |
| GO:0032432 | actin filament bundle | 1.57E-03 | 0.0127 |
| GO:0005801 | cis-Golgi network | 1.61E-03 | 0.0127 |
| GO:0042641 | actomyosin | 1.81E-03 | 0.0127 |
| GO:0047485 | protein N-terminus binding | 3.94E-03 | 0.0473 |
| GO:0015629 | actin cytoskeleton | 8.58E-03 | 0.0129 |

GO, Gene Ontology; FDR, false discovery rate.

**Figure S1. Manhattan-style plots showing the estimates of local genetic correlation between ALS and autoimmune disorders**

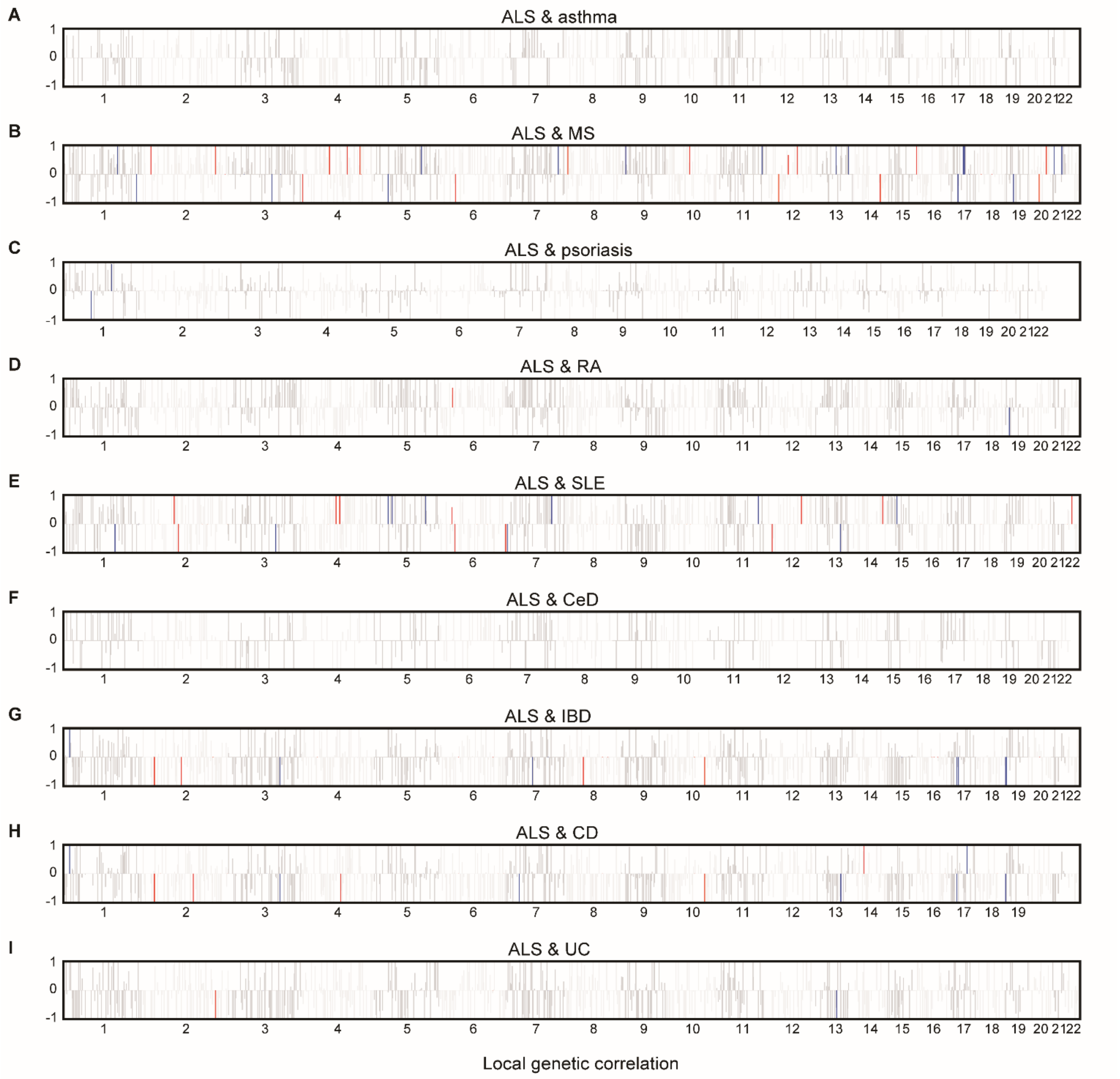


No regions with significant local genetic correlation were identified after Bonferroni correction. Regions with nominally significant correlation (P <0.05) were shown as blue (odd chromosome) and red (even chromosome) lines. Y axis denotes the genetic correlation. Plots were generated using ρ-HESS with default parameters. The figure is suggested to view online for higher resolution.

**Figure S2. Conditional Manhattan plots showing risk loci for ALS conditional on each autoimmune disease**

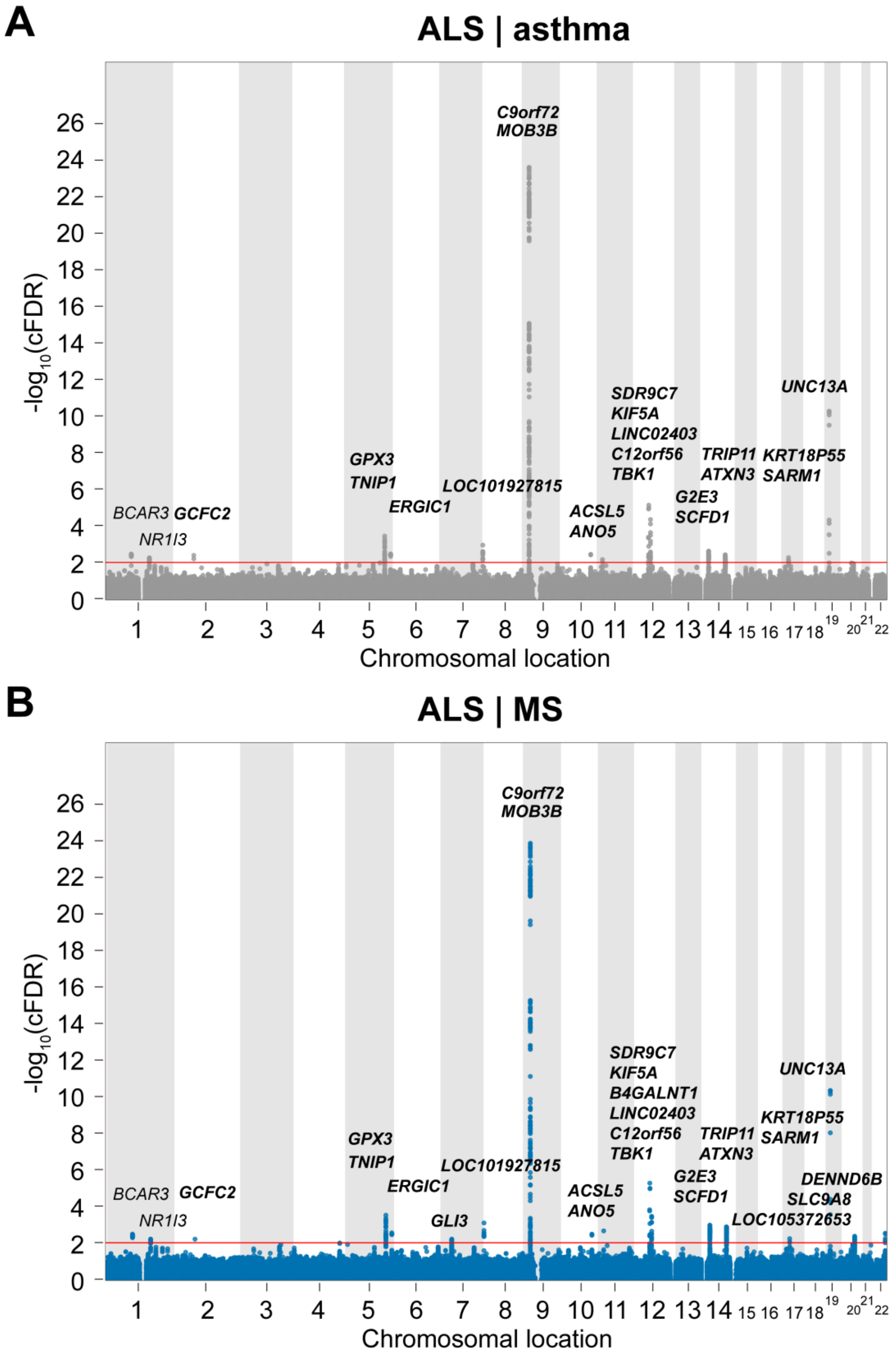

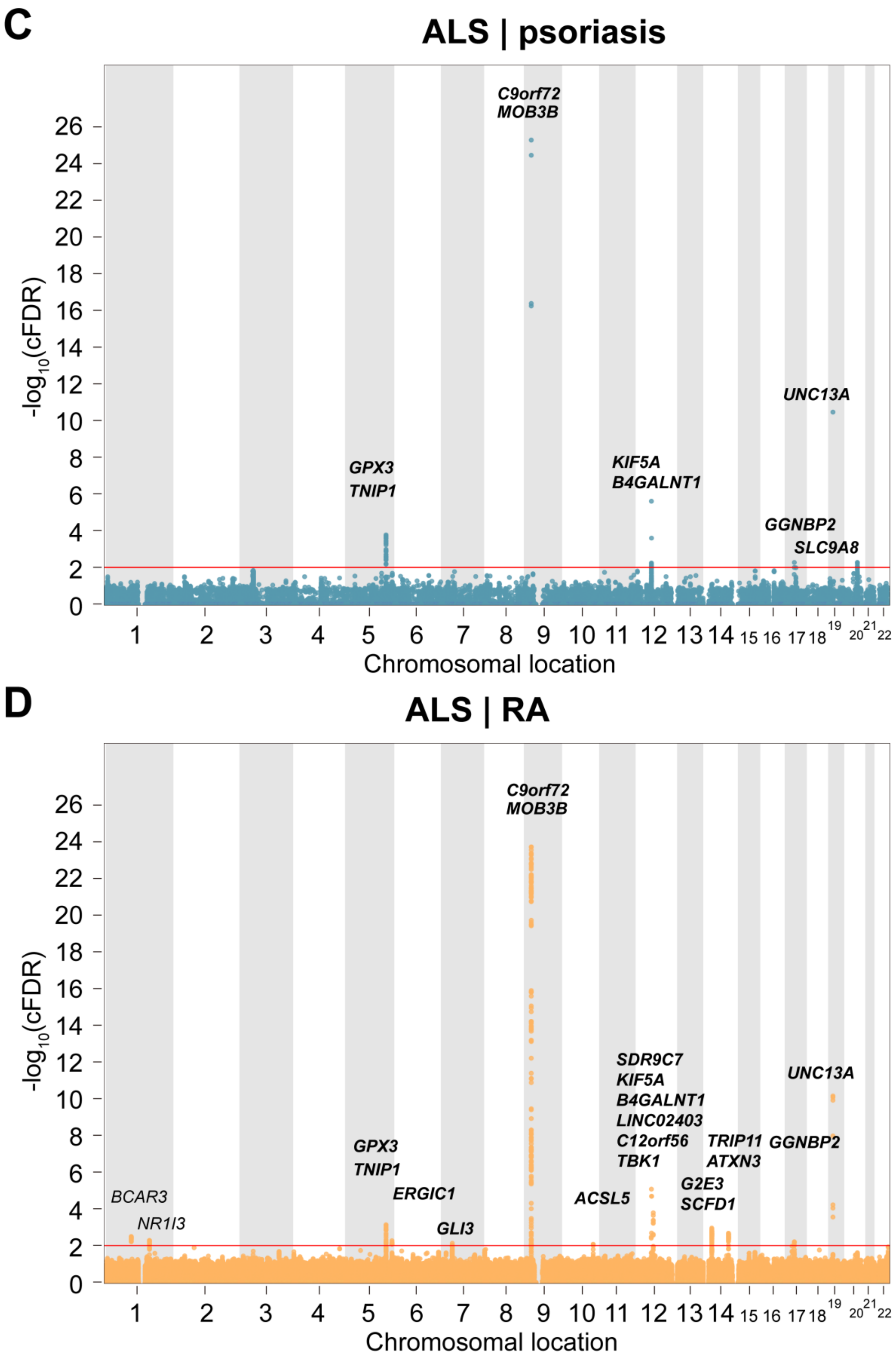
C
ALS | psoriasis
-log10(cFDR)
C9orf72
MOB3B
UNC13A
GPX3
TNIP1
KIF5A
B4GALNT1
GGNBP2
SLC9A8
Chromosomal location
D
ALS | RA
-log10(cFDR)
C9orf72
MOB3B
SDR9C7
KIF5A
B4GALNT1
LINC02403
C12orf56
TBK1
UNC13A
TRIP11
ATXN3
GGNBP2
GPX3
TNIP1
ERGIC1
GLI3
BCAR3
NR1I3
ACSL5
G2E3
SCFD1
Chromosomal location

E

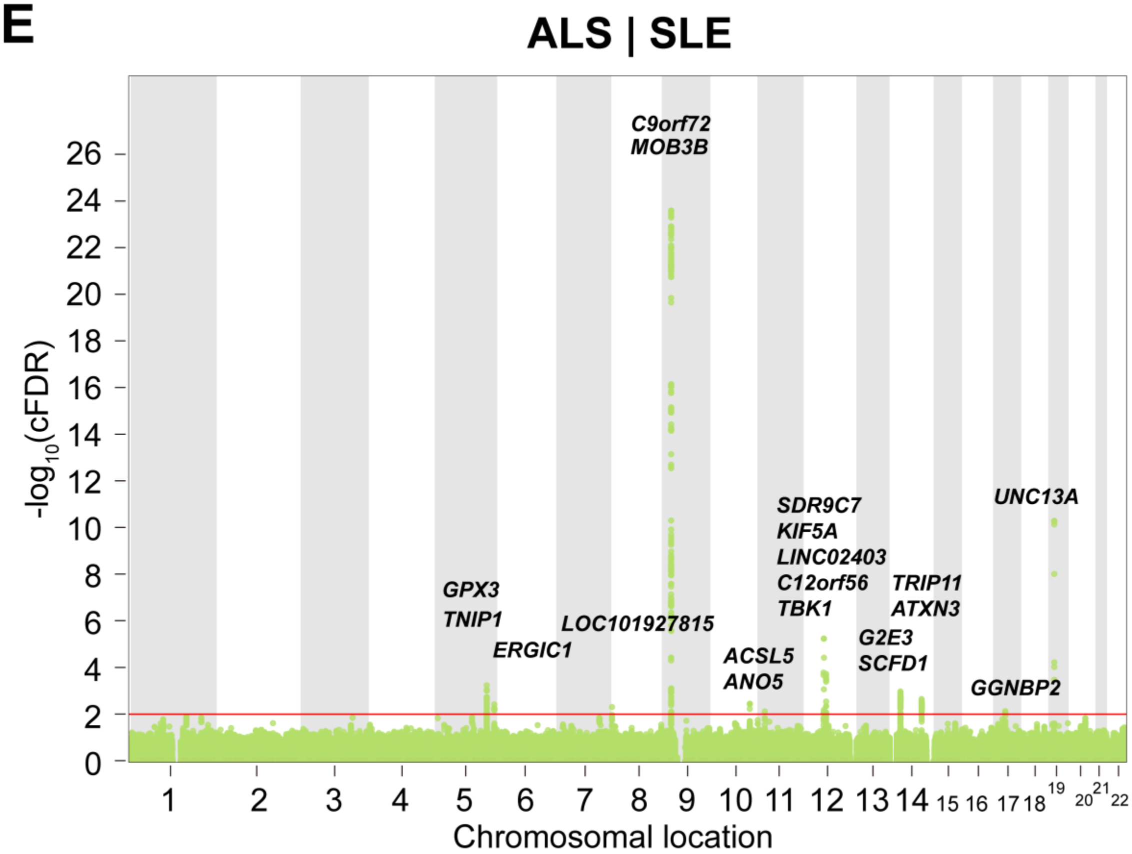
ALS | SLE
-log10(cFDR)
C9orf72
MOB3B
SDR9C7
KIF5A
LINC02403
C12orf56
TBK1
UNC13A
GPX3
TNIP1
ERGIC1
LOC101927815
ACSL5
ANO5
TRIP11
ATXN3
G2E3
SCFD1
GGNBP2
Chromosomal location

F

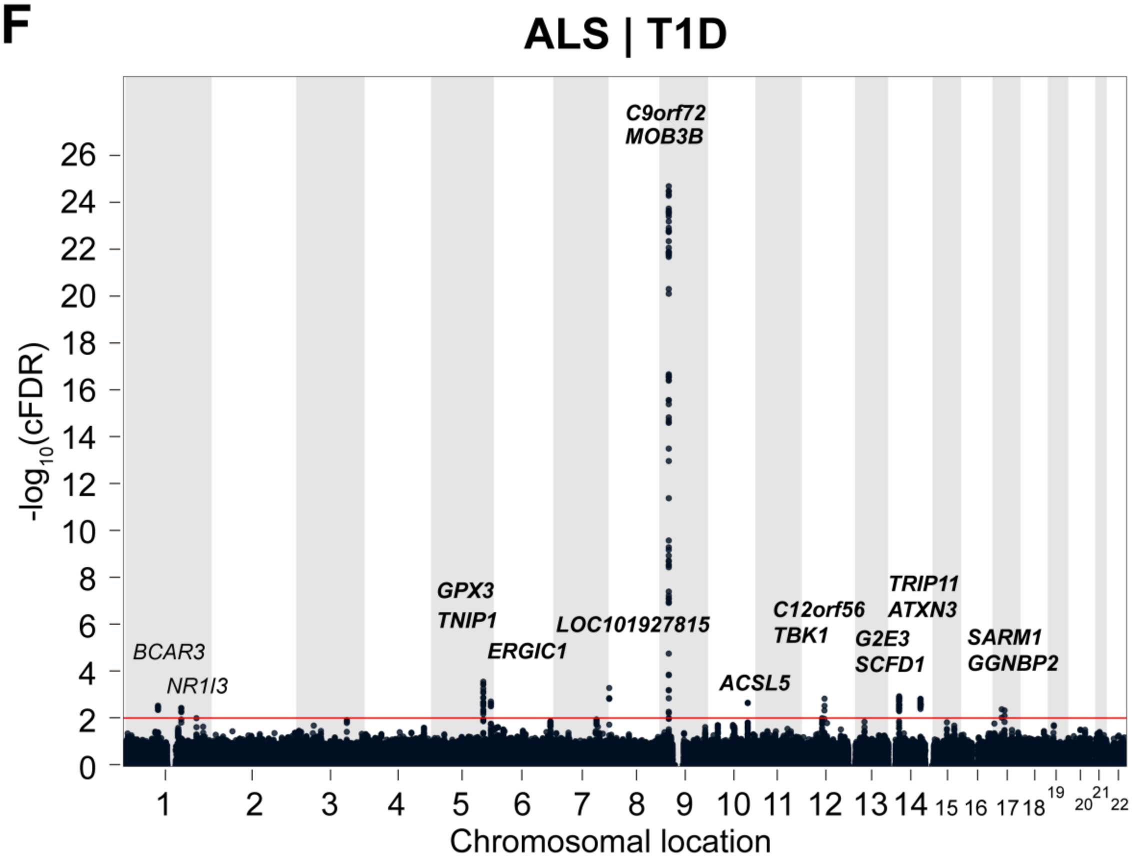
ALS | T1D
-log10(cFDR)
C9orf72
MOB3B
GPX3
TNIP1
ERGIC1
LOC101927815
BCAR3
NR1I3
ACSL5
C12orf56
TBK1
TRIP11
ATXN3
G2E3
SCFD1
SARM1
GGNBP2
Chromosomal location

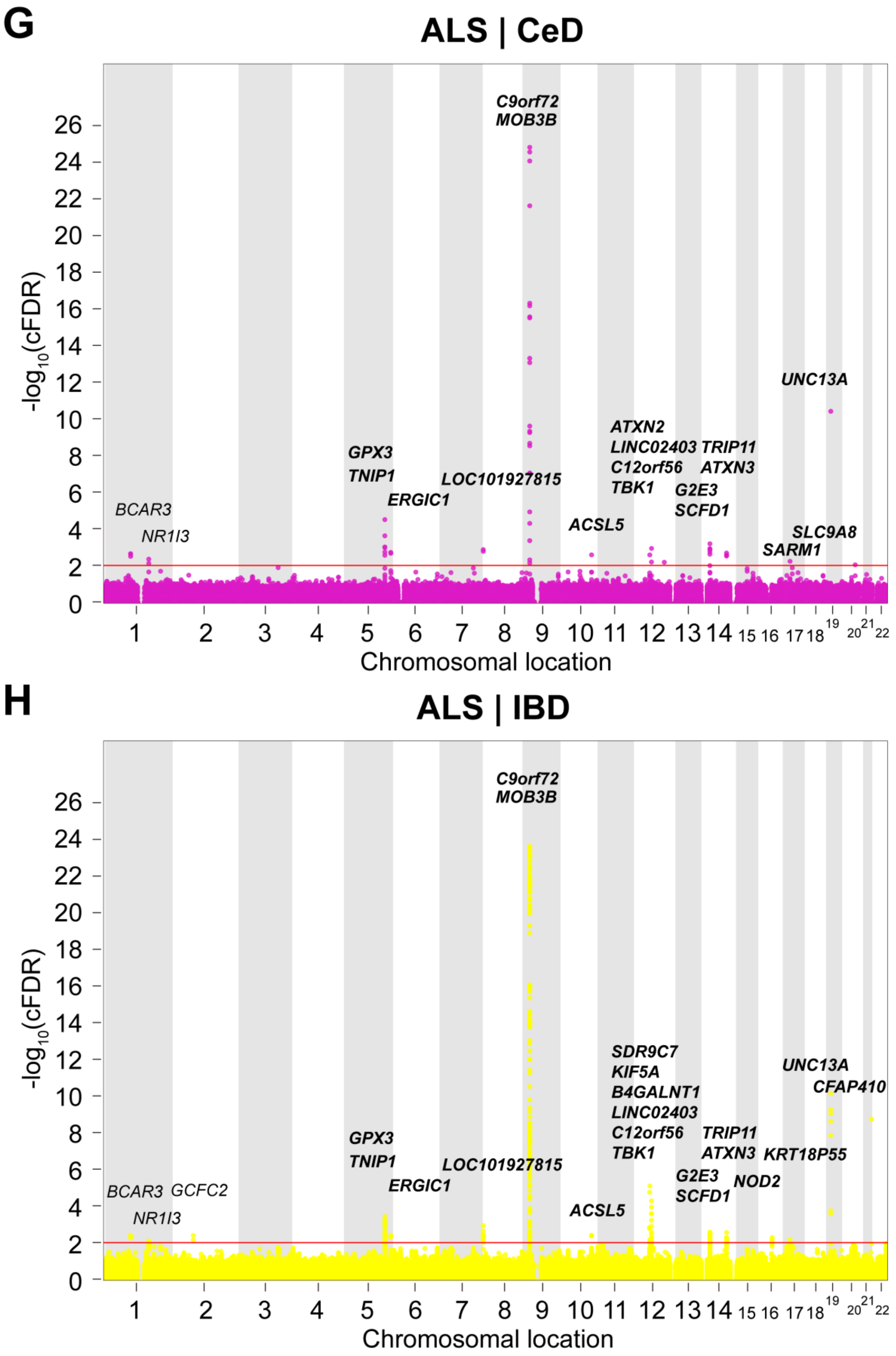
G
ALS | CeD
-log10(cFDR)
Chromosomal location
C9orf72
MOB3B
UNC13A
ATXN2
LINC02403
C12orf56
TBK1
TRIP11
ATXN3
G2E3
SCFD1
GPX3
TNIP1
ERGIC1
LOC101927815
ACSL5
BCAR3
NR1I3
SLC9A8
SARM1
H
ALS | IBD
-log10(cFDR)
Chromosomal location
C9orf72
MOB3B
SDR9C7
KIF5A
B4GALNT1
LINC02403
C12orf56
TBK1
UNC13A
CFAP410
TRIP11
ATXN3
KRT18P55
G2E3
SCFD1
NOD2
GPX3
TNIP1
ERGIC1
LOC101927815
ACSL5
BCAR3
GCFC2
NR1I3

I

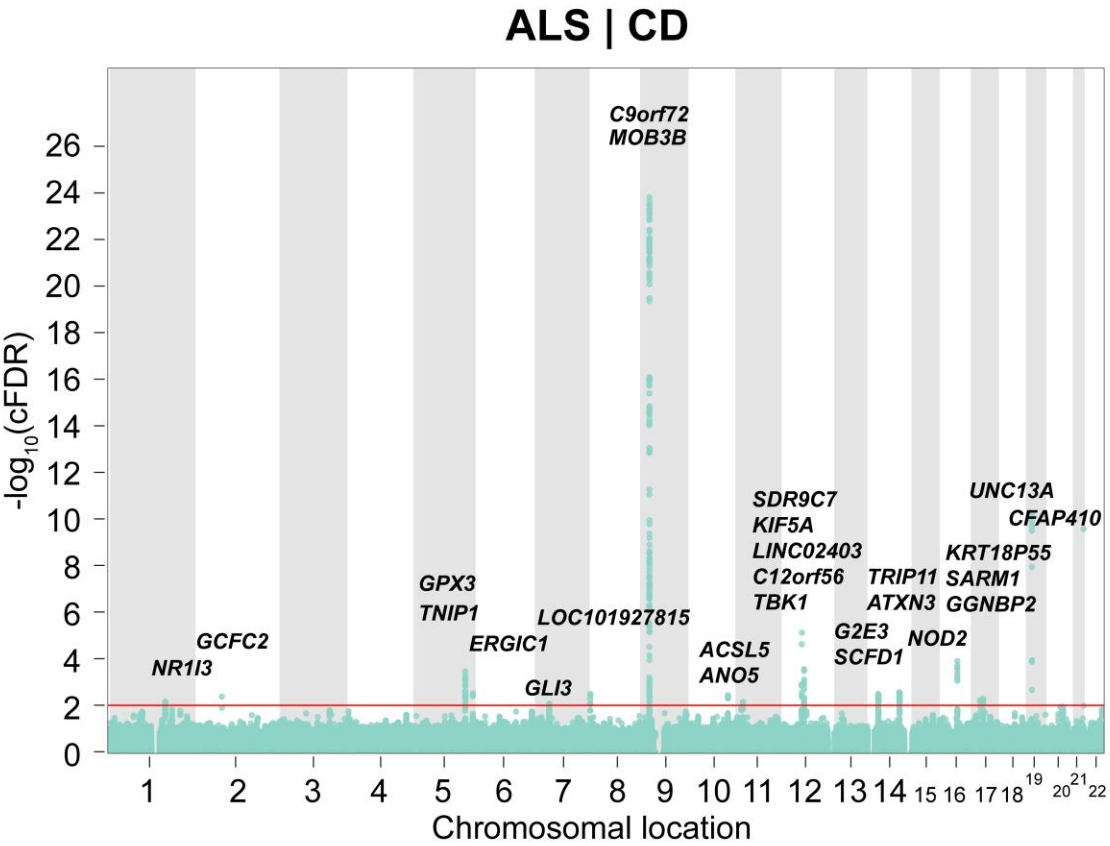
ALS | CD
-log10(cFDR)
Chromosomal location
C9orf72
MOB3B
NR1I3
GCFC2
GPX3
TNIP1
ERGIC1
GLI3
LOC101927815
ACSL5
ANO5
SDR9C7
KIF5A
LINC02403
C12orf56
TBK1
G2E3
SCFD1
TRIP11
ATXN3
NOD2
KRT18P55
SARM1
GGNBP2
UNC13A
CFAP410

J

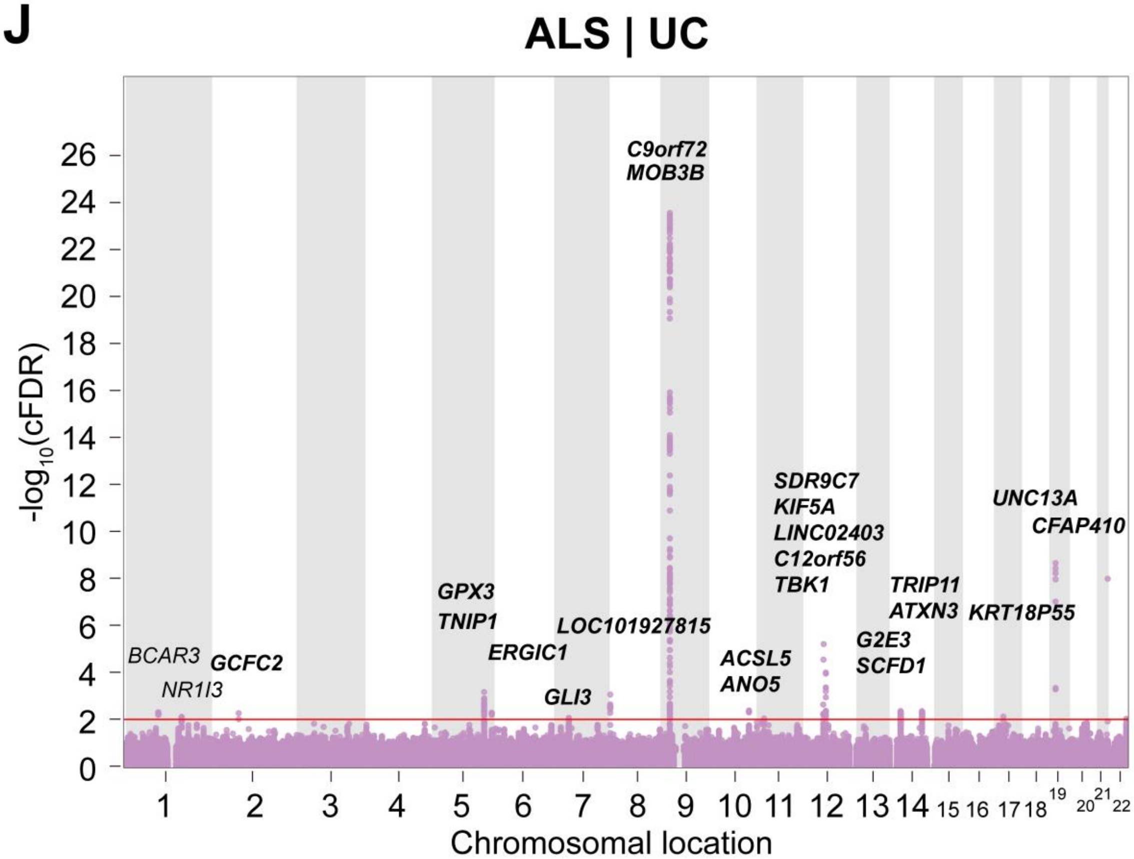
ALS | UC
-log10(cFDR)
Chromosomal location
C9orf72
MOB3B
BCAR3
NR1I3
GCFC2
GPX3
TNIP1
ERGIC1
GLI3
LOC101927815
ACSL5
ANO5
SDR9C7
KIF5A
LINC02403
C12orf56
TBK1
G2E3
SCFD1
TRIP11
ATXN3
KRT18P55
UNC13A
CFAP410

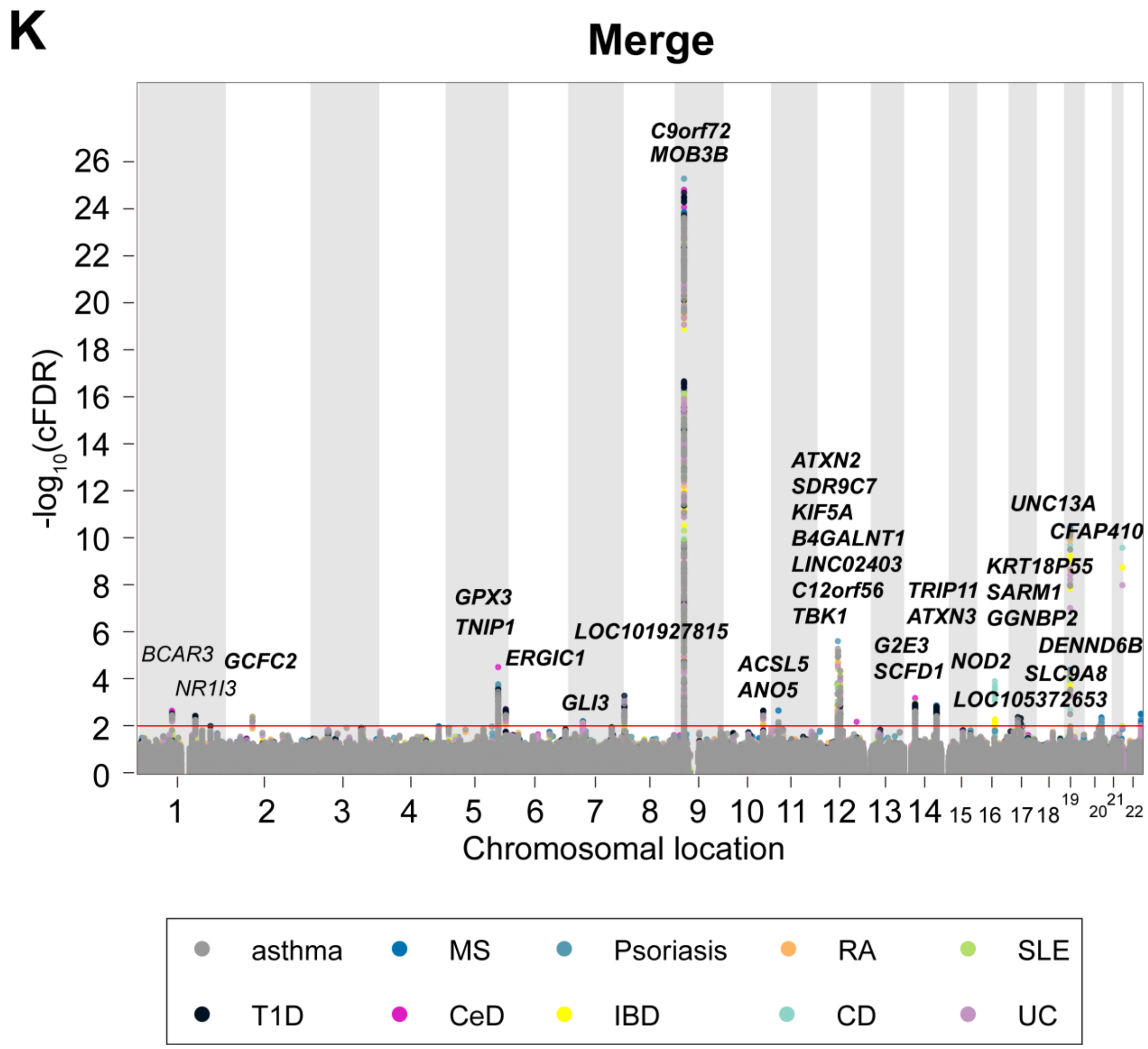


The horizontal line represents the significant threshold (conditional FDR < 0.01). The figure is suggested to view online for higher resolution.

**Figure S3. Regional association plots for shared risk loci between ALS and each autoimmune disease**

A

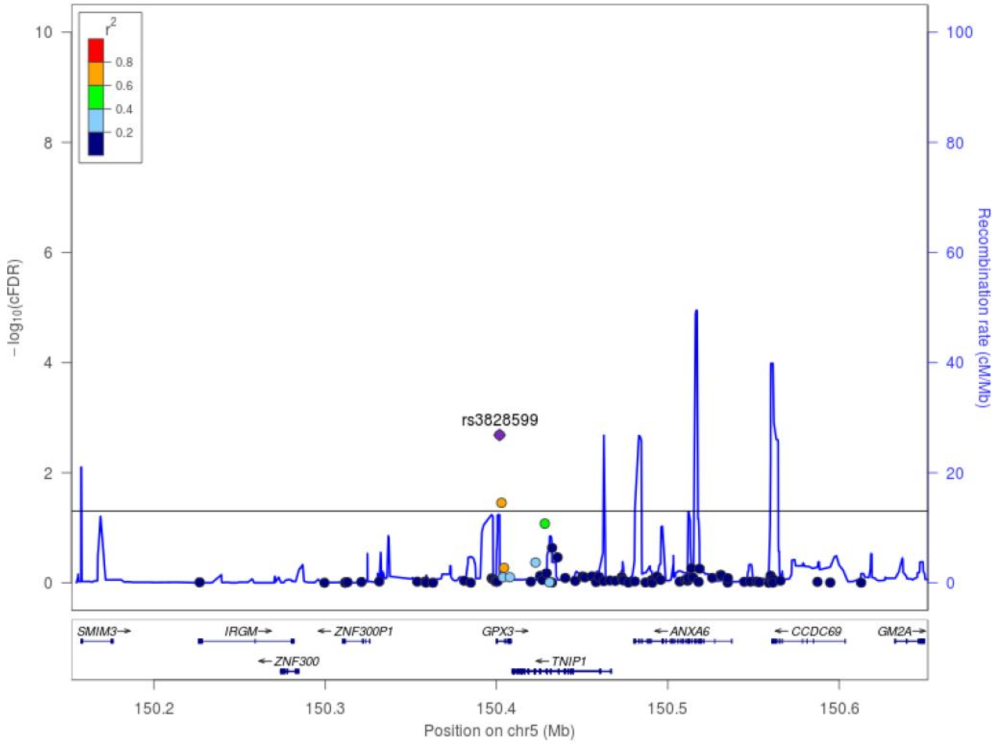


B

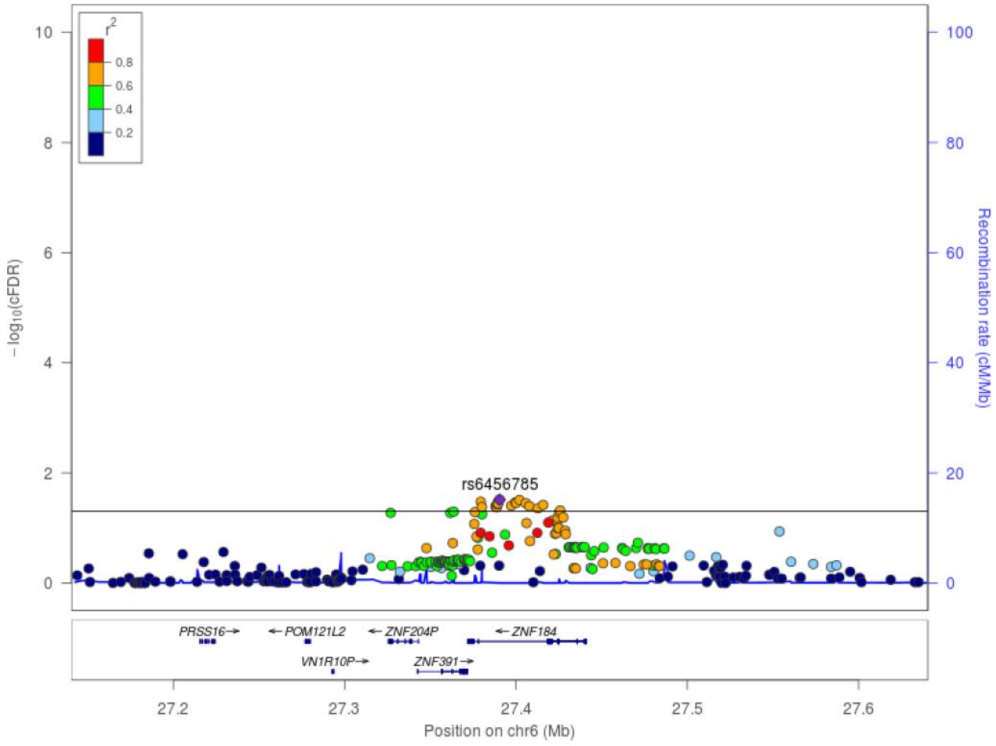

**C**

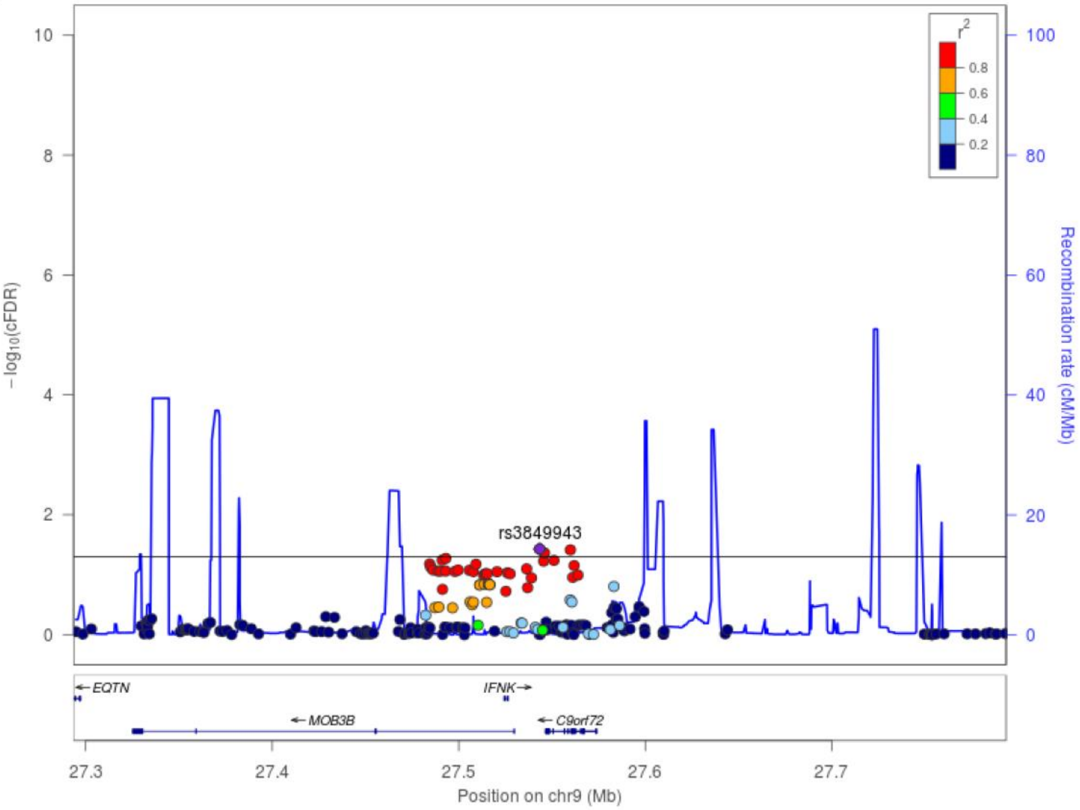
r2
0.8
0.6
0.4
0.2
rs3849943
−log10(cFDR)
Recombination rate (cM/Mb)
EQTN
IFNK
MOB3B
C9orf72
27.3
27.4
27.5
27.6
27.7
Position on chr9 (Mb)

**D**

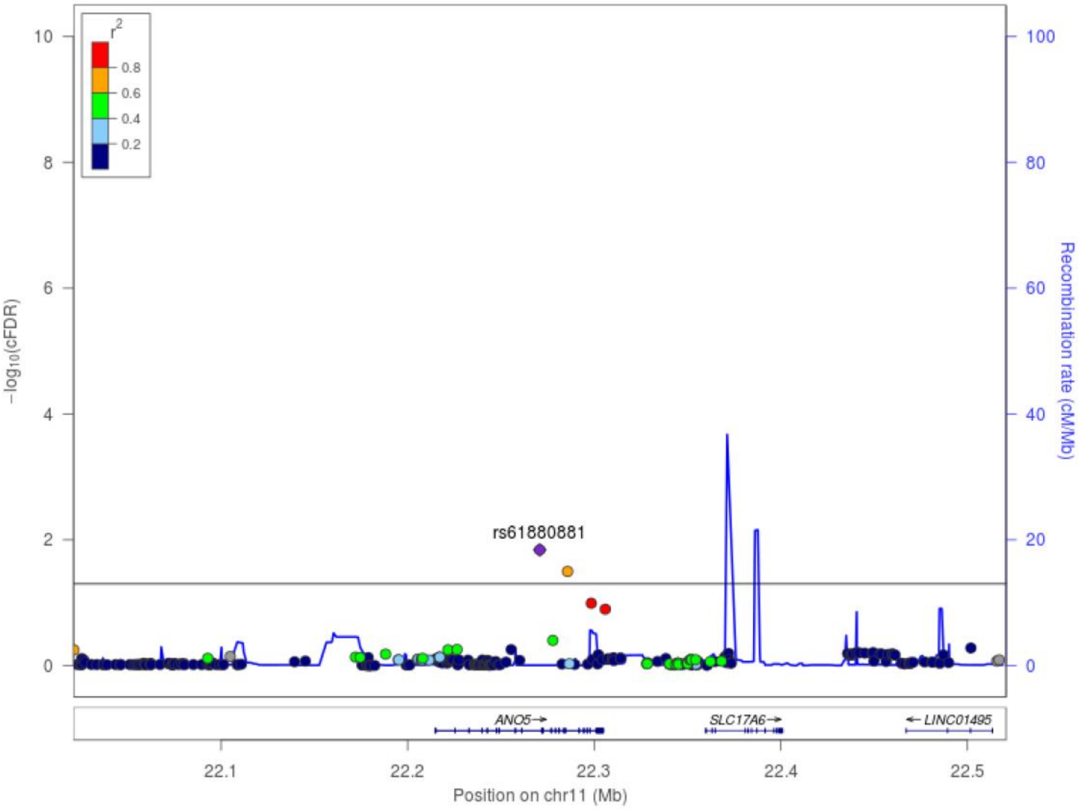
r2
0.8
0.6
0.4
0.2
rs61880881
−log10(cFDR)
Recombination rate (cM/Mb)
ANO5
SLC17A6
LINC01495
22.1
22.2
22.3
22.4
22.5
Position on chr11 (Mb)

E

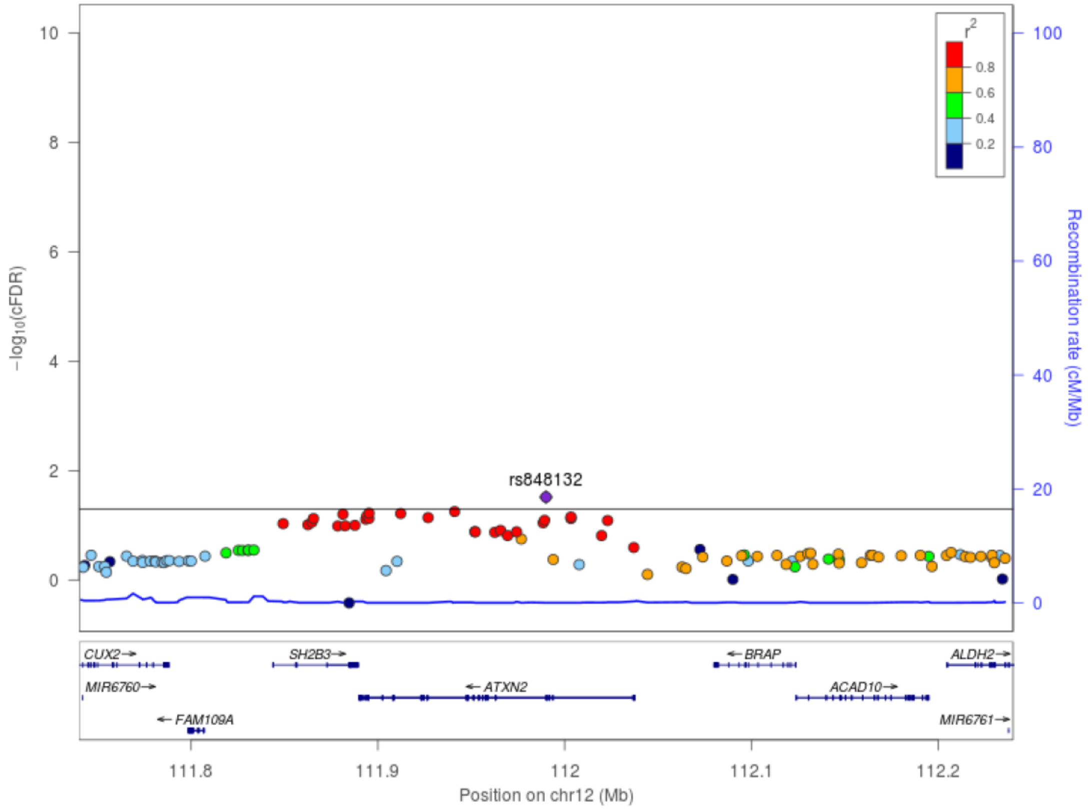
rs848132
−log10(cFDR)
Recombination rate (cM/Mb)
r2
0.8
0.6
0.4
0.2
CUX2→
SH2B3→
←BRAP
ALDH2→
MIR6760→
←ATXN2
ACAD10→
←FAM109A
MIR6761→
111.8
111.9
112
112.1
112.2
Position on chr12 (Mb)

F

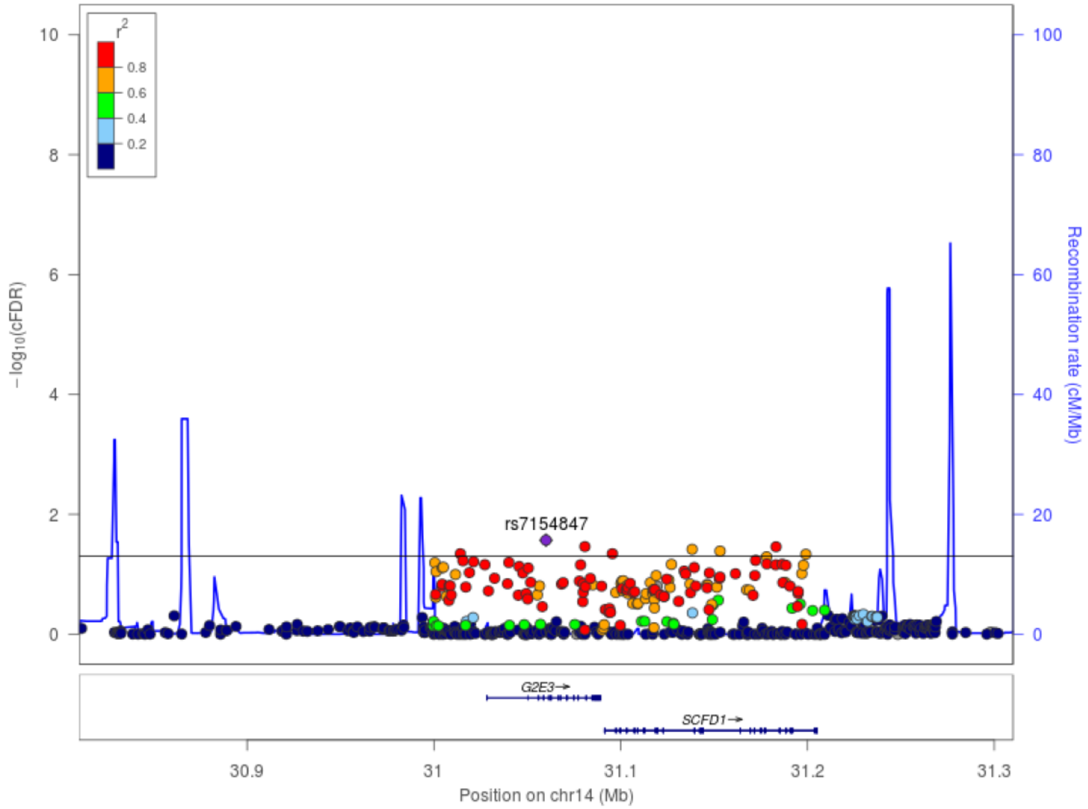
rs7154847
−log10(cFDR)
Recombination rate (cM/Mb)
r2
0.8
0.6
0.4
0.2
G2E3→
SCFD1→
30.9
31
31.1
31.2
31.3
Position on chr14 (Mb)

G

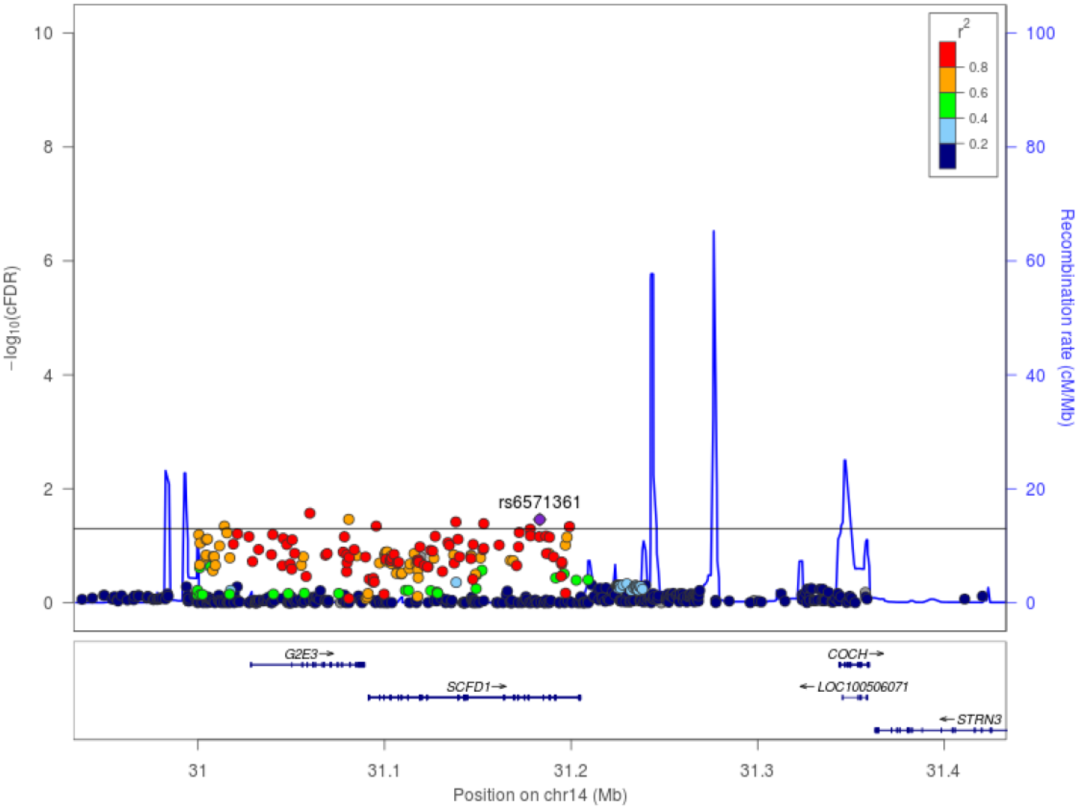
r2
0.8
0.6
0.4
0.2
−log10(cFDR)
Recombination rate (cM/Mb)
rs6571361
G2E3
SCFD1
COCH
LOC100506071
STRN3
Position on chr14 (Mb)
31
31.1
31.2
31.3
31.4

H

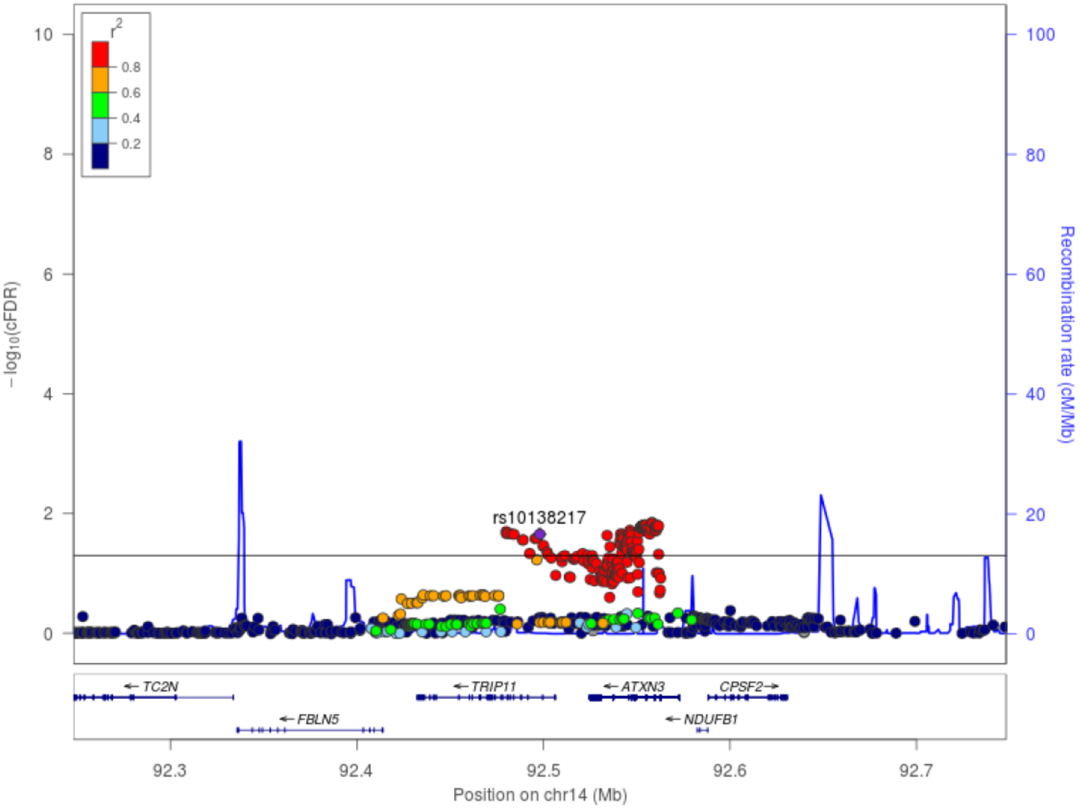
r2
0.8
0.6
0.4
0.2
−log10(cFDR)
Recombination rate (cM/Mb)
rs10138217
TC2N
FBLN5
TRIP11
ATXN3
NDUFB1
CPSF2
Position on chr14 (Mb)
92.3
92.4
92.5
92.6
92.7

I

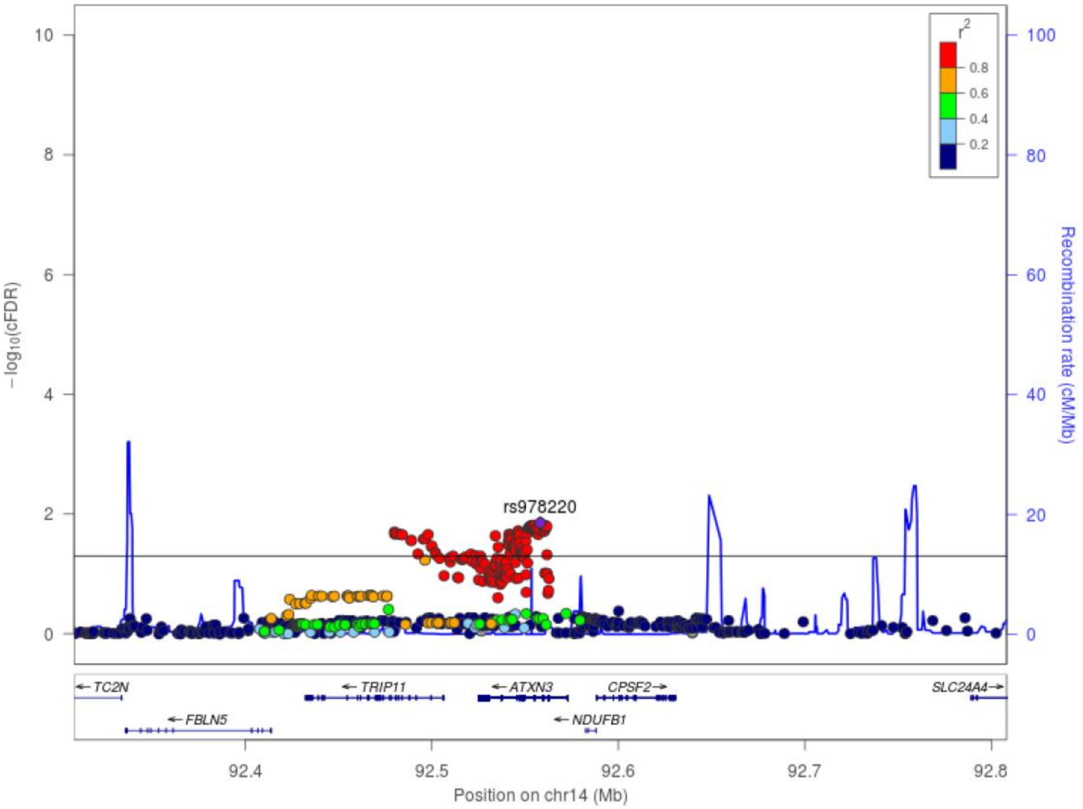
r²
0.8
0.6
0.4
0.2
rs978220
−log10(cFDR)
Recombination rate (cM/Mb)
TC2N
TRIP11
ATXN3
CPSF2
SLC24A4
FBLN5
NDUFB1
Position on chr14 (Mb)

J

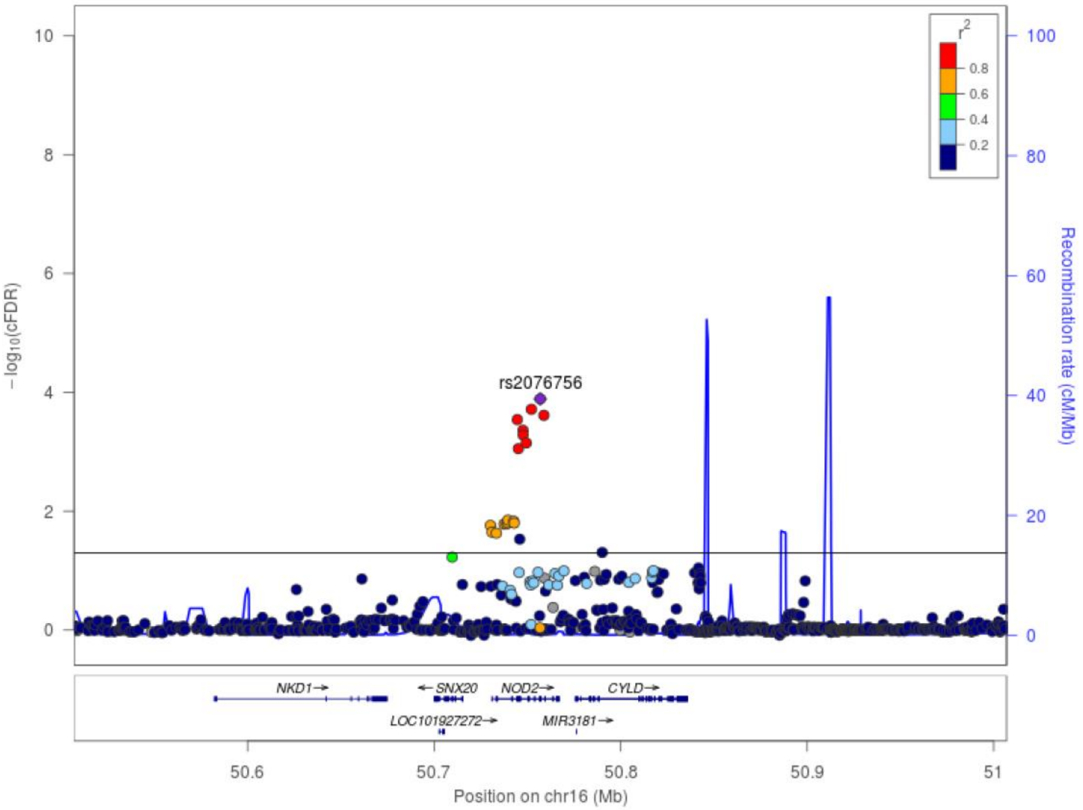
r²
0.8
0.6
0.4
0.2
rs2076756
−log10(cFDR)
Recombination rate (cM/Mb)
NKD1
SNX20
NOD2
CYLD
LOC101927272
MIR3181
Position on chr16 (Mb)

**K**

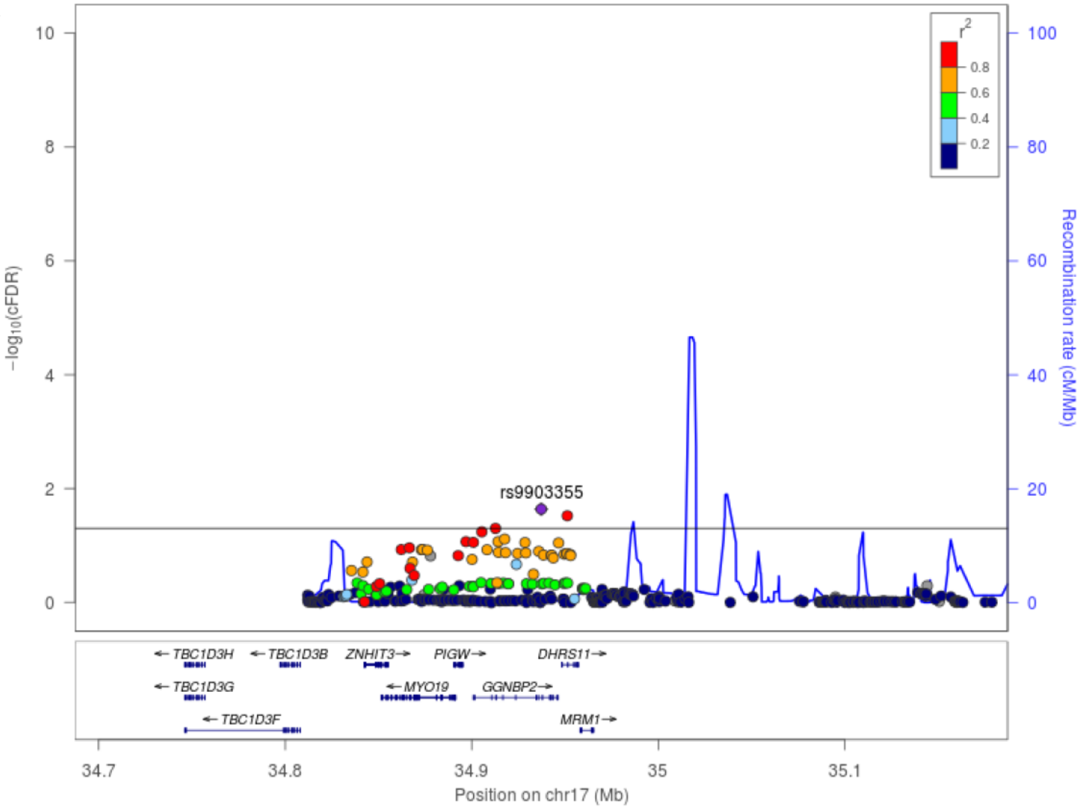

rs9903355
−log10(cFDR)
Recombination rate (cM/Mb)
r2
0.8
0.6
0.4
0.2
TBC1D3H
TBC1D3B
ZNHIT3
PIGW
DHRS11
TBC1D3G
MYO19
GGNBP2
TBC1D3F
MRM1
34.7
34.8
34.9
35
35.1
Position on chr17 (Mb)


**L**

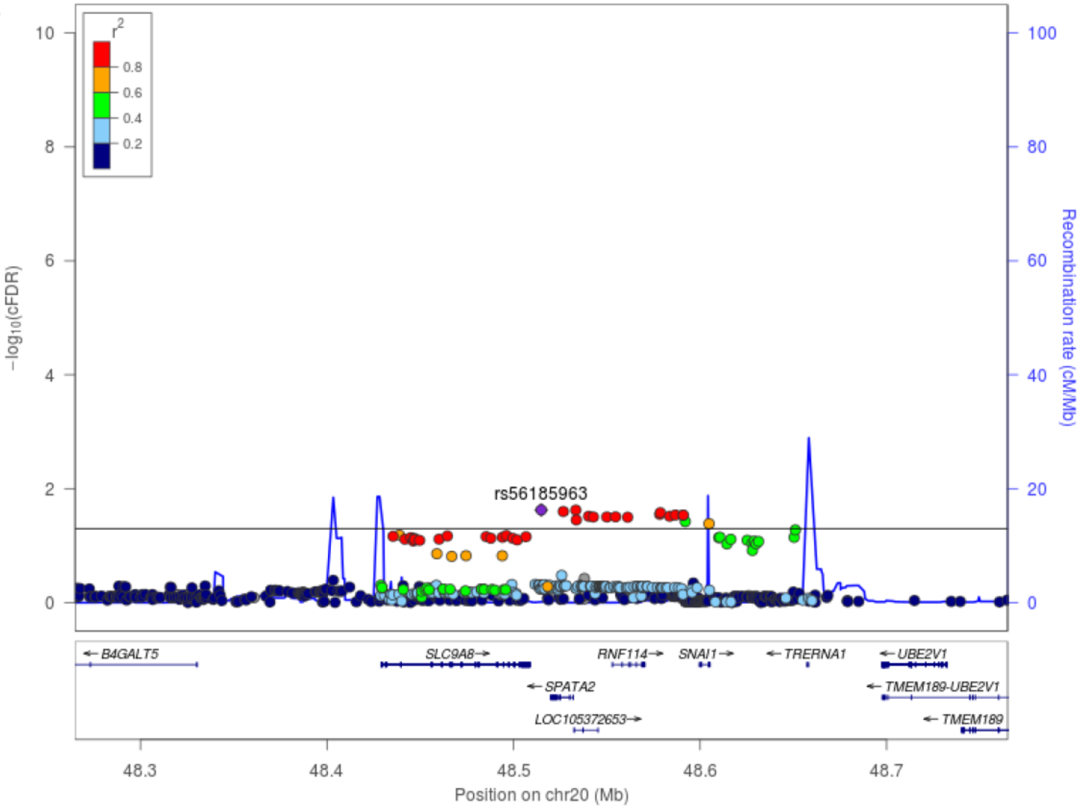

rs56185963
−log10(cFDR)
Recombination rate (cM/Mb)
r2
0.8
0.6
0.4
0.2
B4GALT5
SLC9A8
RNF114
SNAI1
TRERNA1
UBE2V1
SPATA2
TMEM189-UBE2V1
LOC105372653
TMEM189
48.3
48.4
48.5
48.6
48.7
Position on chr20 (Mb)

M

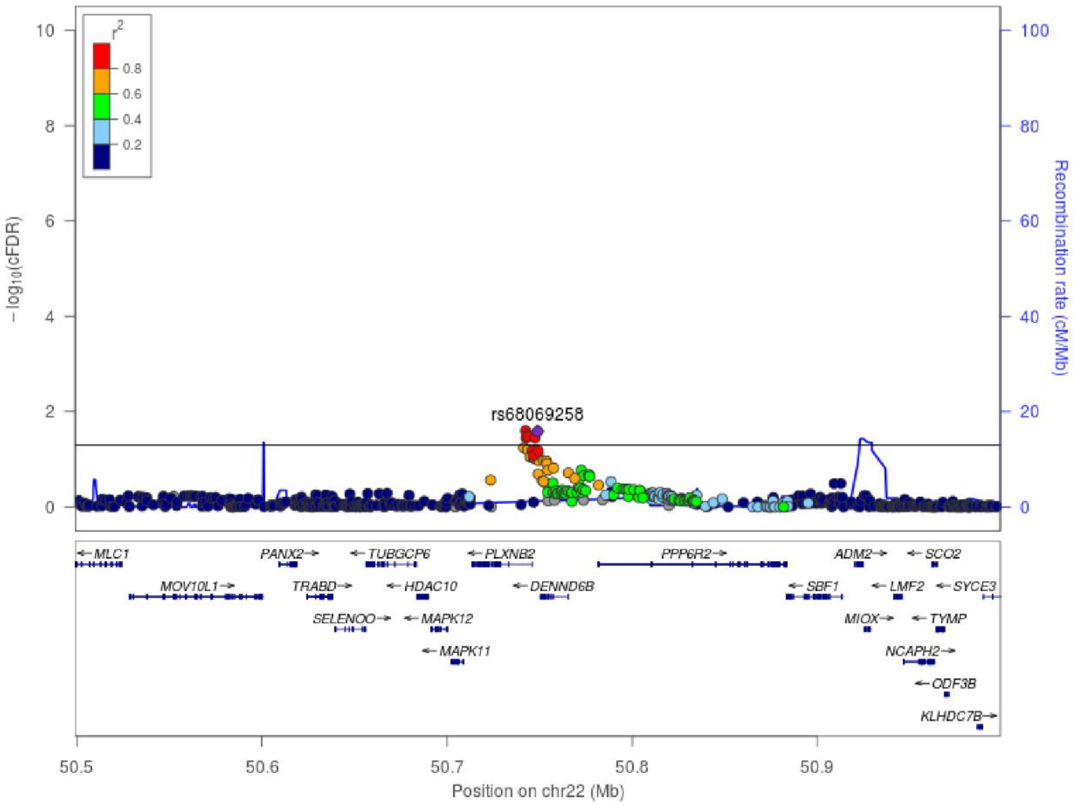


The plots were generated using LocusZoom with default parameters. The horizontal line represents the significant threshold (conditional FDR < 0.05). The linkage disequilibrium was calculated using reference data derived from the 1000 Genomes Project European population. A flanking region of 250kb around target SNP was plotted.
