# Supplementary material for: Genome-wide genetic links between amyotrophic lateral sclerosis and autoimmune diseases
Source: BMC Med. 2021 Feb 5;19:27. doi: 10.1186/s12916-021-01903-y (PMC7863260; doi:10.1186/s12916-021-01903-y)
Supplement: Supplementary file 2 — Additional file 2. Supplementary Methods. [file 12916_2021_1903_MOESM2_ESM.pdf]

Additional file 2: Supplementary Methods

Genomic Control

The empirical null distribution in genome-wide association studies (GWAS) is sometimes inflated due to population stratification, cryptic relatedness, or deflated due to over-correction of test statistics. We applied a genomic control method leveraging only intergenic SNPs, which are likely depleted for true associations and could provide a robust estimate of true null effects. First, we annotated the SNPs to genic (5'UTR, exon, intron, 3'UTR) and intergenic regions using ANNOVAR. We converted all P values to Z scores and then estimated the genomic inflation factor λ_{GC} for each trait. λ_{GC} was calculated as the median Z score squared divided by the expected median of a chi-square distribution with one degree of freedom. Then all P values were divided by λ_{GC} for genomic control adjustment. After this, we pruned the SNPs by removing SNPs in linkage disequilibrium (LD) (r^2>0.2 within 250kb) based on 1000 Genomes Project LD structure using plink –clump functionality.

QQ plots

Quantile-quantile plots compare a nominal probability distribution against an empirical distribution, and leftward deflection of the observed distribution reflects enrichment of low P values. Specifically, we computed the empirical cumulative distribution of nominal P values of ALS for all SNPs and subsets of SNPs with significance level below the indicated cutoffs in each autoimmune disease ($-\log_{10}(P) \geqslant 0$, $-\log_{10}(P) \geqslant 1$, $-\log_{10}(P) \geqslant 2$, $-\log_{10}(P) \geqslant 3$), corresponding to $P \leqslant 1$, $P \leqslant 0.1$, $P \leqslant 0.01$, $P \leqslant 0.001$). To assess for polygenic effects below the standard GWAS significance threshold, we focused on SNPs with nominal $-\log_{10}(P) < 7.3$ (corresponding to $P > 5x10^{-8}$).

Fold enrichment plots

We built fold enrichment plots to quantitatively assess the genetic enrichment between two phenotypes. For a given associated phenotype, enrichment for pleiotropy is present if the degree of deflection from the expected null line is dependent on SNP associations with the second phenotype. Specifically, we computed the empirical cumulative distribution of nominal P values for ALS for all SNPs and for SNPs with significance levels below the indicated cut-offs for each autoimmune disorder ($-\log_{10}(P) \geq 0$, $-\log_{10}(P) \geq 1$, $-\log_{10}(P) \geq 2$, $-\log_{10}(P) \geq 3$ corresponding to $P \leqslant 1$, $P \leqslant 0.1$, $P \leqslant 0.01$, $P \leqslant 0.001$ respectively). The nominal P values ($-\log_{10}(P)$) are plotted on the X-axis, and fold enrichment in ALS as a function of each autoimmune disorder is plotted on the Y-axis. To assess for polygenic effects below the standard GWAS significance threshold, we focused on SNPs with nominal $-\log_{10}(P) < 7.3$ (corresponding to $P > 5 \times 10^{-8}$).

Conditional True Discovery Rate (TDR)

Details of the conditional TDR statistics have been described in the original publication introducing the cFDR method. Briefly, enrichment seen in the fold enrichment plots can be directly interpreted in terms of TDR (equivalent to one minus the False Discovery Rate (FDR)). Specifically, for a given P value cutoff, the FDR is defined as

$$FDR(p) = \pi_0 F_0(p) / F(p), \qquad [1]$$

where π_0 is the proportion of null SNPs, F_0 is the null cumulative distribution functions (cdf), and F is the cdf of all SNPs, both null and non-null. Under the null hypothesis, F_0 is the cdf of the uniform distribution on the unit interval [0,1], so that Eq. [1] reduces to

$$FDR(p) = \pi_0 p / F(p), \qquad [2]$$

The cdf F can be estimated by the empirical cdf $q = N_p/N$, where N_p is the number of SNPs with P values less than or equal to p, and N is the total number of SNPs. Replacing F by q in Eq. [2], we get

$$\text{Estimated FDR}(p) \approx \pi_0 p / q, \qquad [3]$$

which is biased upwards as an estimate of the FDR. Replacing π_0 in Eq. [3] with unity gives an estimated FDR that is further biased upward;

$$q^* \approx p/q \qquad [4]$$

If π_0 is close to one, as is likely true for most GWAS, the increase in bias from Eq. [3] is minimal. The quantity $1 - p/q$, is therefore biased downward, and hence is a conservative estimate of the TDR.

Referring to the formulation of the fold enrichment plots, we see that q* is equivalent to the nominal P value divided by the empirical quantile, as defined earlier. Given the $-\log_{10}$ of the fold enrichment plots we can easily obtain

$$-\log_{10}(q^*) \approx \log_{10}(q) - \log_{10}(p) \qquad [5]$$

demonstrating that the (conservatively) estimated FDR is directly related to an upward shift of the curves in the fold enrichment plots from the expected line x = y, with a larger shift corresponding to a smaller FDR. The estimated TDR can be obtained as 1-FDR. For each P value threshold in the associated trait (e.g. asthma), we calculated the conditional TDR as a function of P value in ALS according to Eq. [5].

Conditional/Conjunctional FDR statistics

Details of the conditional/conjunctional FDR statistics have been described in the original publication introducing the cFDR method. Briefly, the FDR method is based on Bayesian statistics,

and the conditional FDR is the probability of the SNP being null given its P value is as small as or smaller than observed. The conjunctional FDR is an extension of the conditional FDR and is defined as the maximum of the two conditional FDR statistics for a specific SNP. We defined the conjunctional statistics (denoted as $FDR_{Trait1\ \&\ Trait2}$) as the maximum of the conditional FDR in both directions, i.e.

$$FDR_{Trait1\ \&\ Trait2} = \max(FDR_{Trait1\ |\ Trait2}, FDR_{Trait2\ |\ Trait1})$$

based on the combination of P value for the SNP in ALS and each autoimmune disease. The conjunctional statistic allows for the identification of SNPs that are associated with both phenotypes, which minimizes the effect of a single phenotype driving the common association signal.

Conditional/Conjunctional FDR Manhattan plots

To illustrate the localization of the genetic markers associated with ALS conditional on each autoimmune disease, we built the conditional and conjunction FDR Manhattan plots, plotting all SNPs within an LD block concerning their chromosomal location. All SNPs without pruning are shown.
